# PDZ domain-binding motif of Tax sustains T-cell proliferation in HTLV-1-infected humanized mice

**DOI:** 10.1371/journal.ppat.1006933

**Published:** 2018-03-22

**Authors:** Eléonore Pérès, Juliana Blin, Emiliano P. Ricci, Maria Artesi, Vincent Hahaut, Anne Van den Broeke, Antoine Corbin, Louis Gazzolo, Lee Ratner, Pierre Jalinot, Madeleine Duc Dodon

**Affiliations:** 1 Laboratory of Biology and Modeling of the Cell, Ecole Normale Supérieure (ENS) de Lyon, INSERM U1210, CNRS UMR5239, 46 allée d'Italie, Lyon, France; 2 International Center for Infectiology Research, ENS de Lyon, Université Claude Bernard Lyon 1, INSERM U1111, CNRS UMR 5308, 46 allée d'Italie, Lyon, France; 3 Laboratory of Experimental Hematology, Institut Jules Bordet, Université Libre de Bruxelles, Brussels, Belgium; 4 Unit of Animal Genomics, Groupe Interdisciplinaire Génoprotéomique Appliquée (GIGA), Université de Liège, Liège, Belgium; 5 Division of Oncology, Washington University School of Medicine, St Louis, MO, United States of America; University of Illinois at Chicago College of Medicine, UNITED STATES

## Abstract

Human T-cell leukemia virus type 1 (HTLV-1) is the etiological agent of adult T-cell leukemia/lymphoma (ATLL), an aggressive malignant proliferation of activated CD4+ T lymphocytes. The viral Tax oncoprotein is critically involved in both HTLV-1-replication and T-cell proliferation, a prerequisite to the development of ATLL. In this study, we investigated the *in vivo* contribution of the Tax PDZ domain-binding motif (PBM) to the lymphoproliferative process. To that aim, we examined T-cell proliferation in humanized mice (hu-mice) carrying a human hemato-lymphoid system infected with either a wild type (WT) or a Tax PBM-deleted (ΔPBM) provirus. We observed that the frequency of CD4+ activated T-cells in the peripheral blood and in the spleen was significantly higher in WT than in ΔPBM hu-mice. Likewise, human T-cells collected from WT hu-mice and cultivated *in vitro* in presence of interleukin-2 were proliferating at a higher level than those from ΔPBM animals. We next examined the association of Tax with the Scribble PDZ protein, a prominent regulator of T-cell polarity, in human T-cells analyzed either after *ex vivo* isolation or after *in vitro* culture. We confirmed the interaction of Tax with Scribble only in T-cells from the WT hu-mice. This association correlated with the presence of both proteins in aggregates at the leading edge of the cells and with the formation of long actin filopods. Finally, data from a comparative genome-wide transcriptomic analysis suggested that the PBM-PDZ association is implicated in the expression of genes regulating proliferation, apoptosis and cytoskeletal organization. Collectively, our findings suggest that the Tax PBM is an auxiliary motif that contributes to the sustained growth of HTLV-1 infected T-cells *in vivo* and *in vitro* and is essential to T-cell immortalization.

## Author summary

The viral Tax oncoprotein is a critical contributor to the development of adult T-cell leukemia/lymphoma, an aggressive malignant proliferation of T lymphocytes. Tax contains a PDZ domain-binding motif (PBM) that favors the interaction with several cellular PDZ proteins. Here, we compare the *in vivo* involvement of the Tax PBM in humanized mice infected with either a full-length provirus or a Tax PBM-deleted provirus. We observe that the establishment of the sustained lymphoproliferation in the peripheral blood of infected mice is dependent on the Tax PBM. Furthermore, binding of the Tax PBM to the PDZ Scribble protein correlated with perturbations of cytoskeletal organization and cell polarity. In addition, genome-wide transcriptomic analyses strongly suggest that the association of Tax PBM with cellular PDZ proteins results in the expression of several genes involved in proliferation, apoptosis and cytoskeletal organization. Collectively, these results indicate that the Tax PBM is an auxiliary motif that contributes to the growth of HTLV-1 infected T-cells. As a consequence, targeting the PBM/PDZ nodes using small peptides may have the potential to antagonize the Tax-induced lymphoproliferation, offering a novel strategy for the treatment of this disease.

## Introduction

HTLV-1 (Human T-cell leukemia virus, type 1) is the etiological agent of adult T-cell leukemia/lymphoma (ATLL), an aggressive and fatal form of leukemia characterized by the malignant expansion of activated CD4+ T-cells [1]. Among several non-structural regulatory proteins encoded by HTLV-1, Tax, a crucial transcriptional activator of the viral life cycle, exerts pleiotropic effects during the initial stages of the multistep leukemic process [2]. This viral protein modulates the expression of cellular genes leading to the deregulation of T-cell proliferation, perturbing the integrity of cell cycle checkpoints, the DNA damage response and apoptosis pathways [3–6].

Like other viral oncoproteins such as human adenovirus E4-ORF1 and human papillomavirus (HPV) E6, Tax encodes a carboxyl-terminal (ETEV amino acids 350–353) PDZ domain-Binding Motif (PBM) that mediates interactions with a particular group of cellular proteins containing one or several PDZ (PSD95/DLG/ZO-1) domain(s) [7–9]. Many of these PDZ proteins are involved in processes that control cell attachment, cell proliferation, cell polarity and cell signaling [10, 11]. Previous studies have indicated that the interaction of viral oncoproteins with PDZ proteins may play a critical role in the development of malignancies by perturbing the function of these cellular proteins [12, 13]. The HTLV-1 Tax PBM has been shown to associate with several PDZ cellular proteins such as DLG1 (Discs large 1), Scribble, Erbin, TIP-1 (Tax-interacting protein-1) or MAGI-3 (Membrane-associated guanylate kinase-3) in *in vitro* studies [14–16]. One of them, Scribble that acts as a tumor suppressor and a regulator of cell polarity, is highly expressed in activated T-lymphocytes [17, 18].

Interestingly, the absence of this motif in the Tax of HTLV-2, a non-leukemic strain of HTLV, has led to the assumption that the HTLV-1 Tax PBM fulfills an essential function in the leukemic process [19, 20]. Previous studies have shown that the deletion or mutation of the Tax PBM decreases IL2-independent growth of CTLL-2 cells and the Tax transforming activity in a rat fibroblast cell line [19]. More interestingly, Xie et al have reported that PBM is required for virus-mediated T-cell proliferation and genetic instability *in vitro* and for viral persistence in a rabbit infection model [21]. These observations strongly support the hypothesis that the Tax PBM is critically involved in supporting the infectious process, prompting us to *in vivo* evaluate the implication of the PBM in T-cell proliferation. To that aim, we used immunodeficient mice, which display a human hemato-lymphoid system, therein referred to as hu-mice. These hu-mice provide a powerful model for investigating the pathogenesis associated with infection by human lymphotropic viruses [22, 23]. Several studies have previously demonstrated that infection of hu-mice with HTLV-1 recapitulates certain features of ATLL [24–26]. More specifically, our group has demonstrated that HTLV-1 is able to perturb early αβT-cell development in humanized BALB/c Rag2^-/-^γc^-/-^ (BRG) mice [27]. We showed that HTLV-1 infection propelled thymic human T-cell development towards the mature stages and that this effect was dependent on Tax expression.

In this study, we addressed the role of the Tax PBM in hu-mice infected with irradiated cells producing either a wild-type virus (HTLV-1 WT) or a virus characterized by a Tax PBM-deleted (HTLV-1 ΔPBM). In the peripheral blood of WT hu-mice, the proliferation of activated CD4+CD25+ T-cells was significantly higher than in the peripheral blood of ΔPBM hu-mice. Likewise, human T-cells collected from WT hu-mice and cultivated *in vitro* in presence of interleukin-2 were proliferating at a higher level than those from ΔPBM animals. We then showed that the PDZ Scribble protein interacts with Tax in *ex vivo* or *in vitro* T-cells from WT hu-mice, but not in cells from ΔPBM hu-mice. These results underline that the PBM-PDZ association is critical for sustaining HTLV-1-induced T-cell proliferation. Finally, a genome-wide transcriptomic analysis of T-cells from infected hu-mice suggests that this association is involved in the regulation of host genes implicated in cell proliferation, inhibition of apoptosis, cell polarity and cytoskeletal changes.

## Results

### Tax PBM enhances HTLV-1-induced T-cell proliferation in infected hu-mice

**Fig 1:**
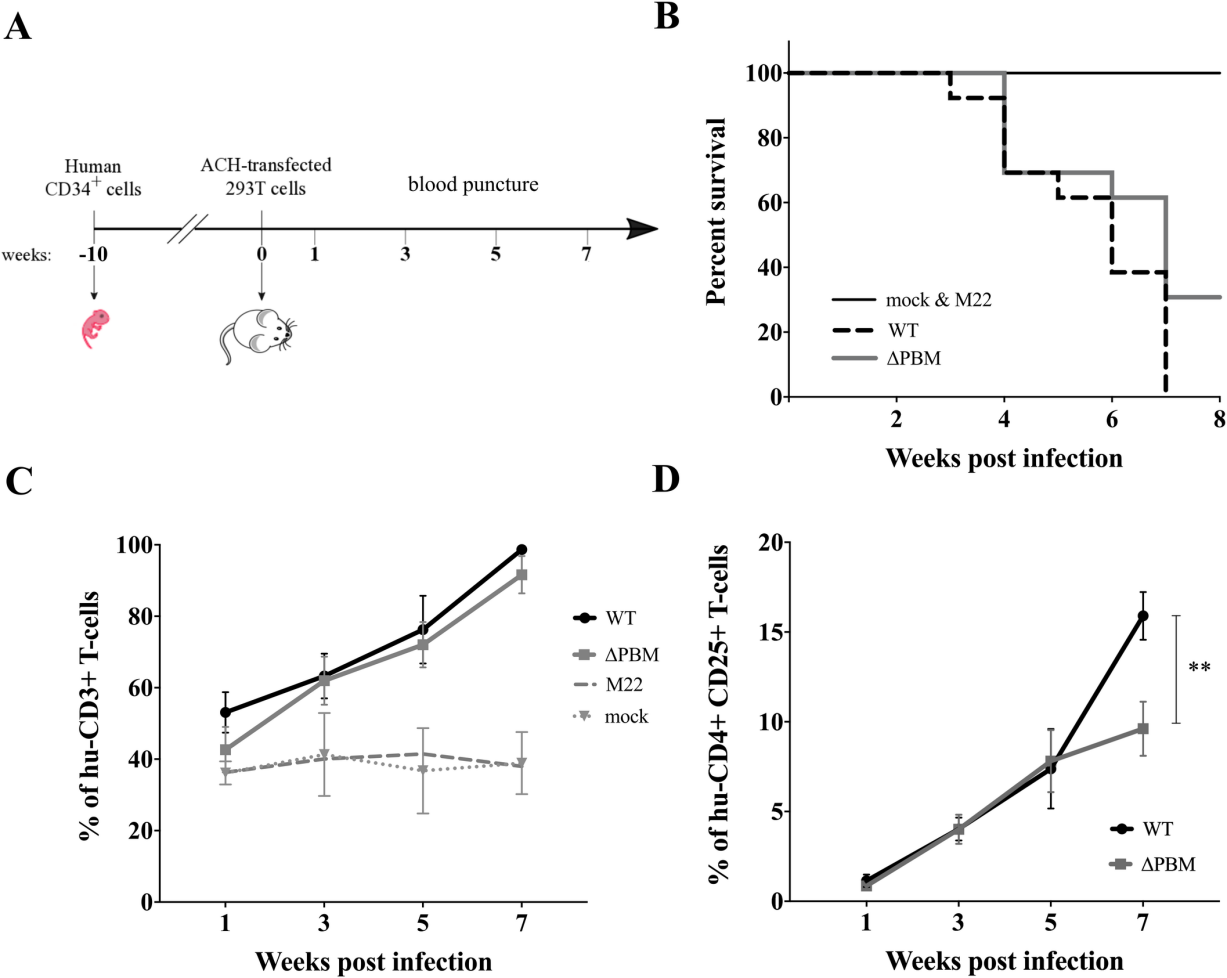
Tax PBM increases HTLV-1-induced proliferation of human CD4+CD25+ T-cells. (**A**) Schematic representation of the procedure for the generation of infected hu-mice: newborn immuno-deficient NSG mice were sub-lethally X-irradiated and intra-hepatically injected with purified huCD34+ stem cells. Ten weeks later, at a time when the human hemato-lymphoid system is established, hu-mice were infected with HTLV-1 by intra-peritoneal inoculation of 293T cells transfected with various ACH plasmids and then X-irradiated. Peripheral blood was collected every two weeks until the sacrifice. (**B**) Representative Kaplan-Meyer analysis of survival of hu-mice infected with ACH-WT (13 animals, dashed line), ACH-ΔPBM (13 animals, grey line), ACH-M22 (3 animals) and 3 mock infected animals (black line). (**C**) Kinetics analysis of the frequency of human CD3+ T-cells among human cells in peripheral blood of WT-(black line), ΔPBM-(grey line); M22 and mock (dashed lines) infected hu-mice. Data are presented as mean± SEM. (**D**) Kinetics analysis of the frequency of human CD4+ CD25+ T-cells among human cells in peripheral blood of 5 WT (black) and 8 ΔPBM (grey) infected hu-mice. To evaluate the frequency, we first gated the hu-CD45+ cells, then the CD3+ cells of hu CD45+cells; then the CD4+/CD8+/ CD25+ of hu-CD3+cells. Statistical difference was calculated with Mann-Whitney *U* test with **, *P* = 0.0093.

To evaluate the role of the Tax PBM *in vivo*, a total of 32 hu-mice were used for this study. Thirteen hu-mice were inoculated with X-irradiated 293T cells previously transfected with either ACH-WT or ACH-ΔPBM molecular clones. Three hu-mice were inoculated with either X-irradiated 293T cells transfected with ACH-M22 (that displays a Tax PBM, but is unable to activate the NF-κB pathway) or untransfected (mock infected). These hu-mice were daily monitored for apparent suffering signs, such as weight loss, back arches and prostrated behavior. Furthermore, a small volume of peripheral blood was eye-harvested from each infected hu-mouse every two weeks starting from one week post-infection (Fig 1A**)** and cytometry analysis was immediately performed to follow the presence of activated human CD25+ T-cells. Accordingly, 8 WT and 5 ΔPBM hu-mice with suffering signs between 3 and 5 weeks after infection were sacrificed. At 7 weeks, the 5 remaining WT hu-mice and 4 ΔPBM hu-mice that exhibited more than 10% of circulating CD25+ T-cells were sacrificed. The 4 surviving ΔPBM hu-mice that did not show any suffering signs were sacrificed two days later together with the 3 ACH M22 and the 3 mock infected animals (Fig 1B**)**.

First, contrary to mock and M22 infected hu-mice, the percentage of circulating hu-CD3+ T-cells of WT and ΔPBM hu-mice increased gradually up to 7 weeks (Fig 1C). This correlated with a significant increase of the frequency of hu-CD45 cells in both WT and ΔPBM infected hu-mice compared to the M22 and mock infected mice (S3 Table). Among the hu-CD3+ T-cells, the frequency of activated CD4+CD25+ T-cells increased gradually up to 5 weeks after infection in the peripheral blood of both WT and ΔPBM hu-mice, while at 7 weeks this percentage was significantly higher in WT than in ΔPBM hu-mice **(**Fig 1D). A low frequency of CD4+CD25+ T-cells was observed in the peripheral blood of ACH-M22 hu-mice as well as in that of mock infected hu-mice **(**S3 Table**)**. We did not observe a significant proliferation of the CD8+ T-cells in the peripheral blood of either group of mice (S3 Table). It is important to note that the Tax transcriptional activity mediated by the WT and ΔPBM proviruses through both CREB/ATF and NF-κB signalling pathways is independent of the Tax PBM (S1 Fig). In addition, it is evident that the PBM is operational only when the NF-κB pathway is functional. Collectively, these data suggest that the PBM is endowed with a sustaining activity of T-cell proliferation.

**Fig 2:**
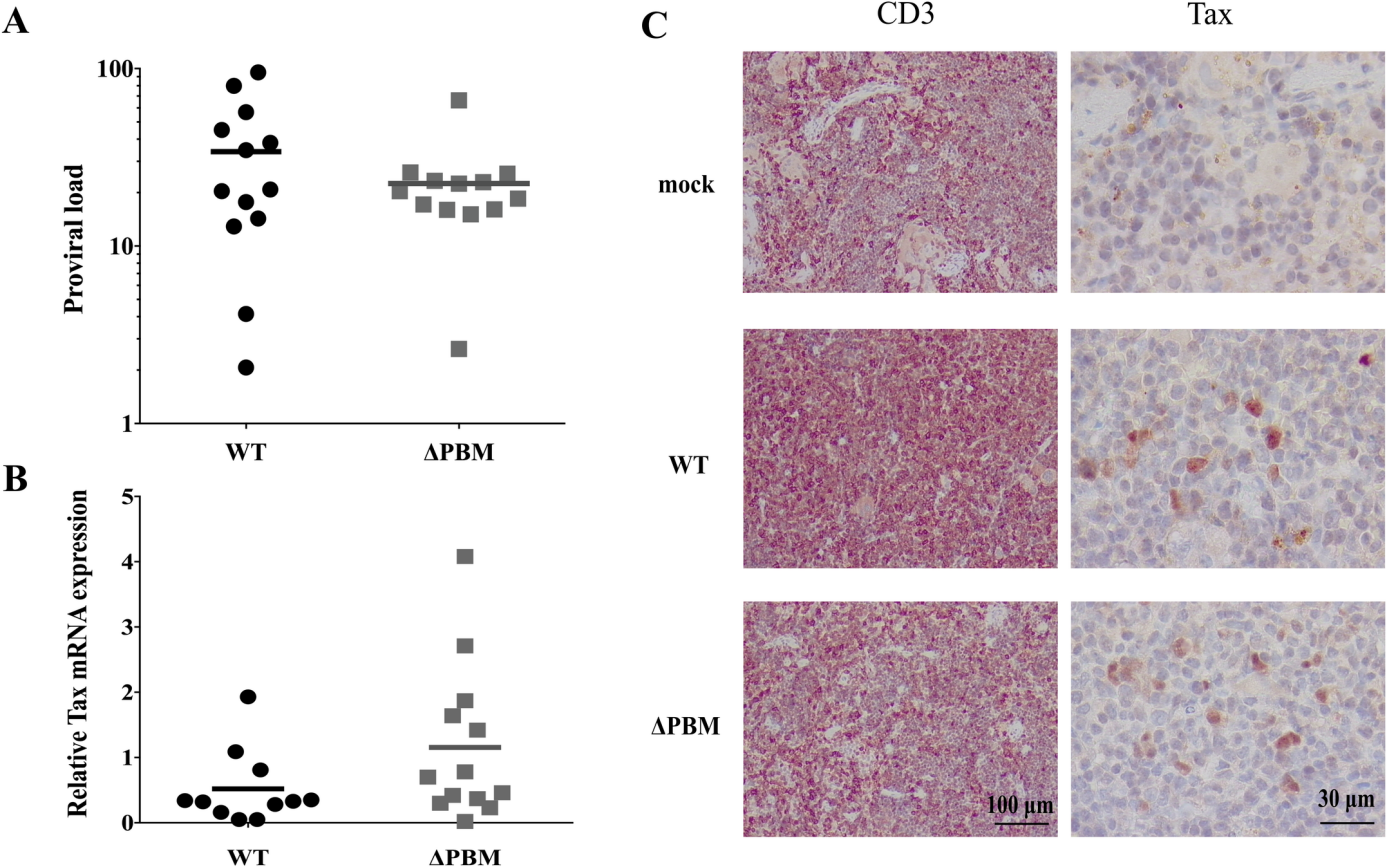
Proviral load and Tax expression in the spleen of WT or ΔPBM infected hu-mice. (**A**) The proviral load in splenocytes from the 2 groups of 13 hu-mice infected with the respective HTLV-1 variants was determined by quantitative PCR and reported as the number of *pX* copies per 100 human cells. Bar represents mean. The Mann Whitney *U* test indicates no statistical difference between the two conditions, *P* = 0.2939. (**B**) Tax mRNA expression in splenocytes isolated from HTLV-1-infected hu-mice with HTLV-1 variants. Levels of Tax mRNA were measured by RT-qPCR; bar represents mean. Mann-Whitney *U* test, *P* = 0.0579. (**C**) Immunohistochemistry of representative sections of spleen of WT and ΔPBM infected hu-mice; staining with CD3 and Tax revealed an infiltration of T-lymphocytes with a nuclear and cytoplasmic localization of Tax.

Examination of sacrificed mice revealed that enlargement of the spleen was the most frequently observed pathological symptom. Splenomegaly was observed in all, but five WT and six ΔPBM hu-mice respectively (S4 Table). Spleen was collected as well as bone marrow and when possible mesenteric lymph nodes. There was no significant difference in the spleen weight between groups (mean of 0.255±0.199 g for WT vs 0.232±0.126 g for ΔPBM hu-mice compared to 0.103±0.037 g for mock and M22 infected hu-mice) (S4 Table). Sequence analysis of genomic DNA prepared from splenocytes of infected hu-mice confirmed the original sequence of the ACH-WT or ACH-ΔPBM molecular clones used for infection (S5 Table). PVL of both WT and ΔPBM hu-mice splenocytes was between 0.1 to 1 copy/cell with no significant difference between groups (Fig 2A; S4 Table). Using high throughput sequencing (HTS)-based mapping of HTLV-1 integration sites, we did not observe significant differences in the number of unique insertion sites (UIS) corresponding to the number of independent clones between WT and ΔPBM hu-mice (S2 Fig). Similar levels of Tax mRNA and protein were detected in CD3+ T-cells from splenocytes of both WT and ΔPBM infected hu-mice (Fig 2B and 2C and S3 Fig). There was no correlation between Tax mRNA levels and the weight of the spleens.

**Fig 3:**
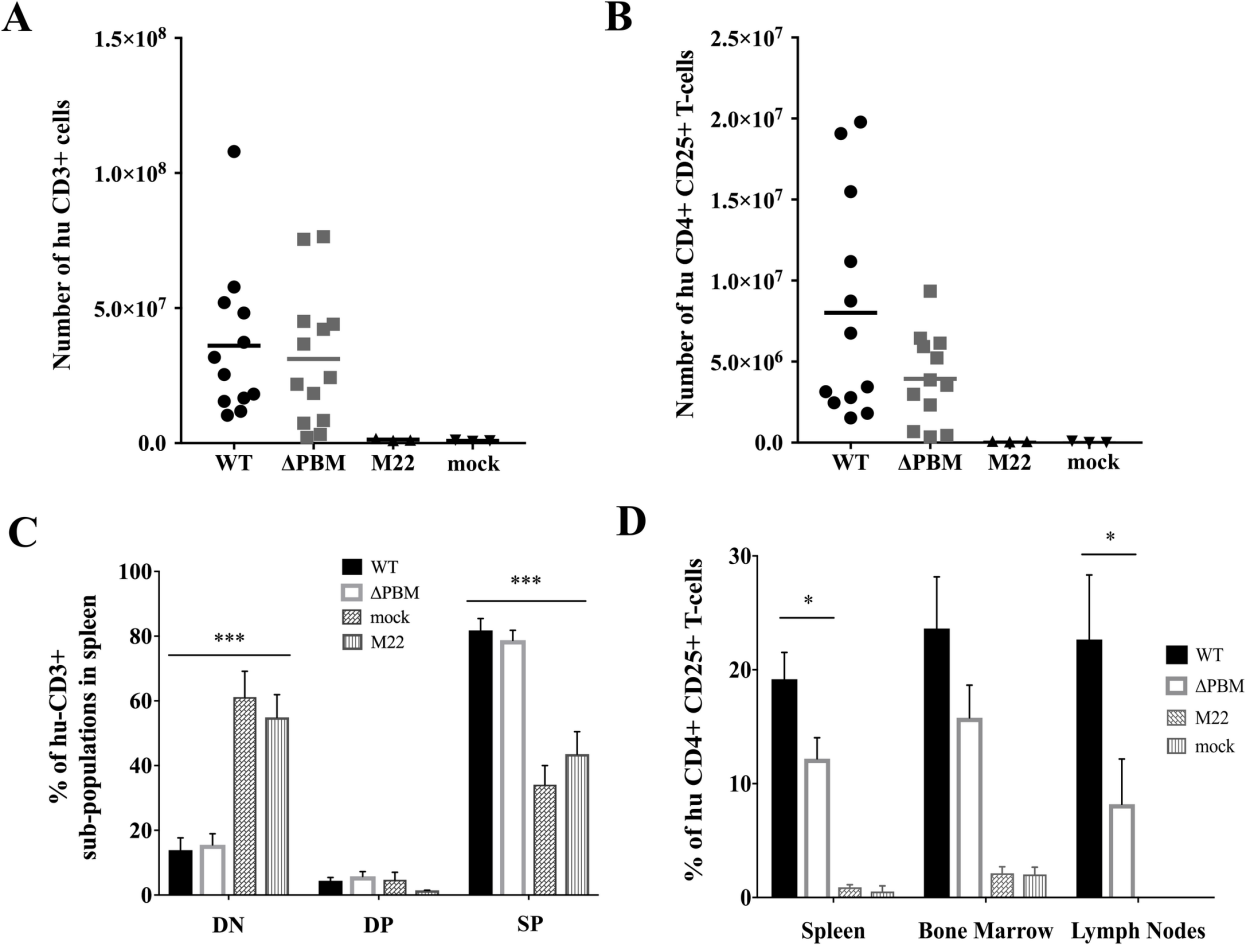
Tax PBM increases the frequency of human CD4+CD25+ T-cells in lymphoid organs of infected hu-mice. (**A**) Number of human CD3+ T-cells among human cells in the spleen of infected mice (WT, n = 13; ΔPBM, n = 13; M22, n = 3; mock, n = 3). **(B)** Summary of human CD4+ CD25+ T-cell expansion in the spleen of infected mice. (**C**) Composite data from 13 WT (black), 13 ΔPBM (white), 3 M22 (grey) and 3 mock (crossed) infected mice showing the frequency of human CD3+ T-cells subpopulations: DN (CD4-CD8-), DP (CD4+CD8+) and SP4 and SP8 in the spleen of infected hu-mice. Data are represented as mean± SEM. Statistical significance was determined using the ANOVA test with ***, *P* < 0.005. (**D**) Frequency of human CD4+ CD25+ T-cells among human cells in lymphoid organs from infected mice (WT, n = 13; ΔPBM, n = 13; M22, n = 3; mock, n = 3). Lymph nodes were not detected in M22 and mock infected hu-mice. Data are represented as mean± SEM. Statistical difference was calculated with Mann-Whitney *U* test with *, *P* < 0.05.

As shown in Fig 3 and S6 Table, the number and the percentage of CD3+ T-cells collected from the spleen of WT and ΔPBM infected hu-mice were similar and higher than those observed in ACH M22 and mock infected hu-mice. Remarkably, a comparative analysis of the T-cell subpopulations (DN: CD4-CD8-; DP: CD4+CD8+, and SP: CD4+ or CD8+) among these CD3+ T cells in both WT and ΔPBM infected hu-mice revealed a low percentage of the DN T-cells and a high percentage of the SP T-cell population (among which CD4+ T-cells dominated over CD8+ T-cells), in sharp contrast with the distribution profile of these subpopulations in ACH M22 and mock infected hu-mice (Fig 3C and S4 Fig). Interestingly, even if similar degrees of splenomegaly (S4 Table) that correlated with similar numbers of CD3+ T-cells in the spleen (Fig 3A) were detected, it remains that the number as well as the percentage of CD4+CD25+ T-cells among human splenocytes were higher in WT than in ΔPBM hu-mice (Fig 3B–3D). Likewise, lymph nodes and bone marrow collected from WT hu-mice showed a higher frequency of CD4+ CD25+ T-cells than those from ΔPBM-hu mice. As indicated above, such a difference also observed in the peripheral blood of animals sacrificed at 7 weeks suggests that the seeding of the periphery by CD4+CD25+ T-cells homing from lymphoid organs is more efficient for WT hu-mice than for ΔPBM hu-mice. Altogether, these results indicate that hu-mice are providing an appropriate environment for the proliferation of human T-cells infected with either HTLV-1 WT or ΔPBM proviruses. Overall, they underline that the HTLV-1 Tax PBM is acting as an *in vivo* auxiliary motif in the HTLV-1-induced proliferation of infected human T-cells.

### Tax PBM mislocalizes Scribble in T-cells from WT, but not from ΔPBM hu-mice and sustains proliferation of WT T-cells

**Fig 4:**
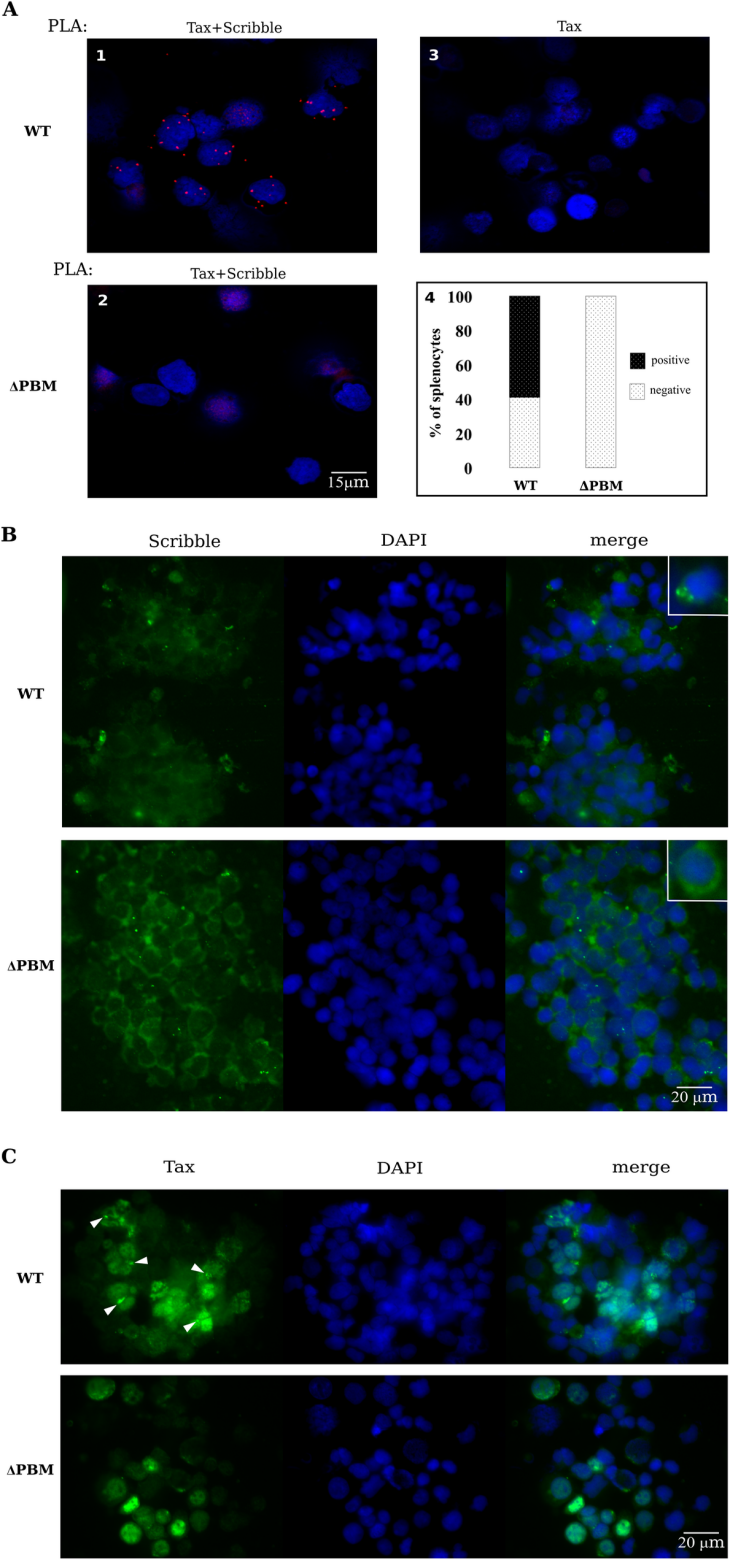
Tax PBM interacts with Scribble and induces its mis-localization *ex vivo*. (**A**) Tax PBM interacts with endogenous Scribble in splenocytes extracted at sacrifice (*ex vivo*) of WT (1, 3) and Δ PBM (2) infected-hu mice. A direct quantification of Tax/Scribble interactions (red dots) performed by *in situ* Proximity Ligation Assay (PLA) is shown in panel 4. Primary anti-Tax, anti-Scribble antibodies were combined with secondary PLA probes (Olink Bioscience). Nuclei are stained in blue (DAPI). Negative control (3) was performed in the absence of anti-Scribble antibodies. (**B-C**) Tax PBM alters subcellular localization of endogenous Scribble in infected hu-mice. Splenocytes collected from WT and ΔPBM infected hu-mice were stained at sacrifice with anti-Scribble (B) and anti-Tax (C), and with DAPI (blue) for nuclear staining. Arrows indicate the presence of condensed aggregates of Tax.

As introduced above, several studies have documented that the Tax PBM mediates interactions with a select group of PDZ-containing proteins [8, 19, 21, 28]. In the present study, we focused our attention on one of them, the Scribble protein, that under physiological conditions is differentially localized throughout polarized T-cells and acts as a tumor suppressor [18]. Indeed, Scribble has been shown to undergo mislocalization in cultured HTLV-1 infected T-cells [14, 15]. We first examined the interaction of Tax with Scribble and the localization of the two proteins in *ex vivo* splenocytes collected immediately from WT and ΔPBM infected hu-mice after their sacrifice, by using the *in situ* Proximity Ligation Assay (PLA) technology. PLA is a reliable readout of the molecular proximity of two endogenous proteins, thereby facilitating the direct observation of individual protein complexes *in situ* [29]. Thus, the presence of at least 4 dots per cell in about 60% of splenocytes from WT hu-mice clearly revealed a direct contact between Tax and Scribble (Fig 4A, panel 1 and 4). In contrast, we did not observe similar interactions in ΔPBM hu-mice (Fig 4A, panel 2). With regards to the intracellular localization of Scribble and Tax in these *ex vivo* splenocytes, immunofluorescent-staining (IF) assays clearly indicated that Scribble was preferentially detected in large polarized aggregates in the cytoplasm of cells from WT mice. In contrast, it was diffusely localized in the cytoplasm and at the plasma membrane of cells from ΔPBM hu-mice (Fig 4B). Concomitantly, Tax was found to be mostly localized in the cytoplasm and also visible in condensed aggregates at the plasma membrane of cells from WT hu-mice (Fig 4C). In contrast, Tax was detected in the nucleus and in the cytoplasm of cells from ΔPBM hu-mice. These data strongly suggest that PBM is associated with the sequestration of Scribble into polarized aggregates of T-cells from WT hu-mice. As mislocalization of Scribble might interfere with its tumor suppressor function, one can postulate that the Tax PBM is implicated in the enhanced T-cell proliferation observed *in vivo* in WT hu-mice.

**Fig 5:**
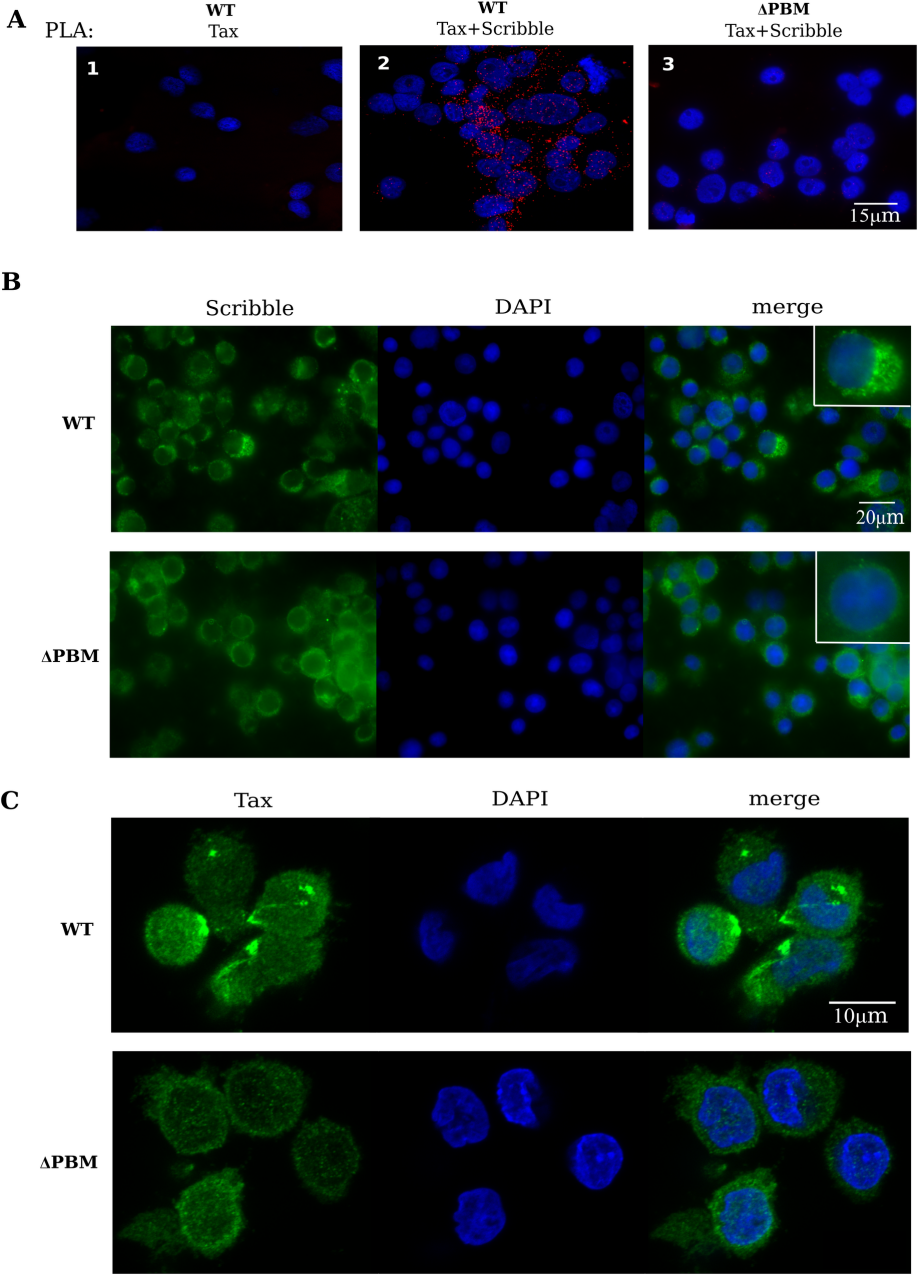
Tax PBM interacts with Scribble and induces its mis-localization *in vitro*. (**A**) Interaction of Tax PBM and endogenous Scribble in cultured T-cells obtained from the spleen of WT and ΔPBM hu-mice, by PLA as described in Fig 4A. (**B**) Subcellular localization of endogenous Scribble in cultured T-cells obtained from the spleen of WT and ΔPBM infected hu-mice. (**C**) Subcellular localization of Tax in cultured T-cells obtained from the spleen of WT and Δ PBM infected hu-mice.

**Fig 6:**
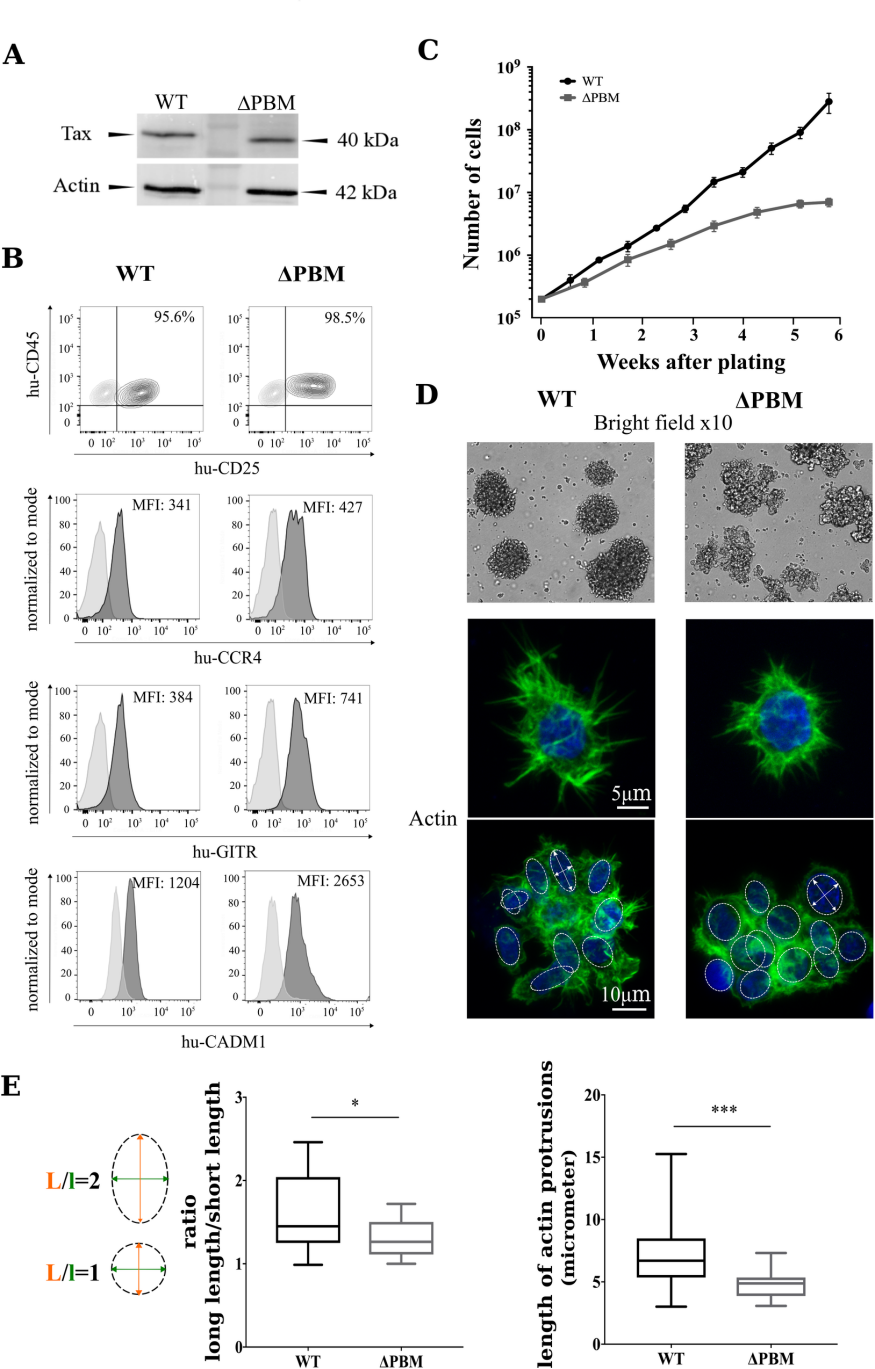
Tax PBM sustains the proliferation of T-cells from HTLV-1 infected hu-mice. (**A**) Expression of Tax in the cultured T-cell lines isolated from WT and ΔPBM infected hu-mice. Western blot analysis was performed using anti-Tax and anti-actin antibodies. (**B**) Phenotypic characterization of the cultured T-cell lines isolated from WT and ΔPBM infected hu-mice by FACS analysis of the CD25, CCR4, GITR and CADM-1 markers. (**C**) Growth curves. Human T-cells (2x10^5^) isolated from the spleen of a WT (black line) or a ΔPBM (grey line) infected hu-mice were cultured in growth medium supplemented with IL2 in 24-well plates. Cells were split as indicated and counted. The mean and sem of each time point was determined from triplicate counts from one of three representative experiments performed at 2-, 5- and 8-months of *in vitro* culture. (**D**) Clumps of WT and ΔPBM T-cells and the actin cytoskeleton shown by IF staining. (**E**) Quantification of the ratio long/short length of the nuclei (Mann-Whitney one-paired: *P* = 0.0267) (left panel), and the length of the protrusions (Mann-Whitney one-paired: *P* = 0.0001) (right panel).

Such a possibility was further investigated in assaying the proliferation of T-cells collected from WT or ΔPBM hu-mice and *in vitro* seeded in growth medium supplemented with IL2. We periodically verified that these T-cells contained integrated copies of the provirus used at infection (S5 Table**).** Interestingly, the patterns of Tax/Scribble interaction (Fig 5A) and subcellular localization of both Tax and Scribble (Fig 5B and 5C) in these cultured human T-cells were identical to those observed in *ex vivo* splenocytes. We also observed that they expressed similar amounts of Tax (Fig 6A). Likewise, periodic FACS analyses of both types of cells revealed the presence of a majority of CD25+, GITR+, CCR4+ and CADM-1+ T-cells (Fig 6B). Interestingly, cell enumeration performed during several weeks showed that WT T-cells were actively proliferating, in contrast to ΔPBM T-cells that displayed a restrained growth (Fig 6C). Overall, it is important to note that, contrary to ΔPBM T-cells, the proliferation of which was regularly in crisis, WT T-cells constantly proliferated and became immortalized. Taken together, these observations proposed that the Tax-PBM is enhancing HTLV-1-mediated T-cell proliferation and is necessary to immortalize T-cells isolated from hu-mice.

It is well known that T-cells infected by HTLV-1 are forming large clumps when cultivated *in vitro* in presence of IL2. We observed that the clumps of WT T-cells were regular and concentric whereas those of ΔPBM T-cells were irregular and eccentric (Fig 6D). These observations are reminiscent of the FACS data showing differences in the size and granularity of *ex vivo* CD4+CD25+ T-cells isolated from the spleens of either WT hu-mice or ΔPBM hu-mice (S5 Fig). Furthermore, the WT T-cells displayed long protrusions (filopods) of actin while ΔPBM T-cells showed shorter actin filopods, suggesting that PBM might be involved in cell migration (Fig 6D). We also observed that in WT cells, the nuclei were oval (ratio L/l = 1.8) while they were spherical in ΔPBM cells, suggesting that the cytoskeleton in WT cells exerts a distortion force on the nuclei (Fig 6D and 6E). In summary, *in vivo* and *in vitro* data indicate that Tax PBM mislocalizes Scribble leading to morphological and cytoskeletal modifications that correlate with a sustained proliferation of WT T-cells.

### Tax PBM impacts transcriptional pathways: A genome-wide transcriptomic analysis of T-cells isolated from infected hu-mice

**Fig 7:**
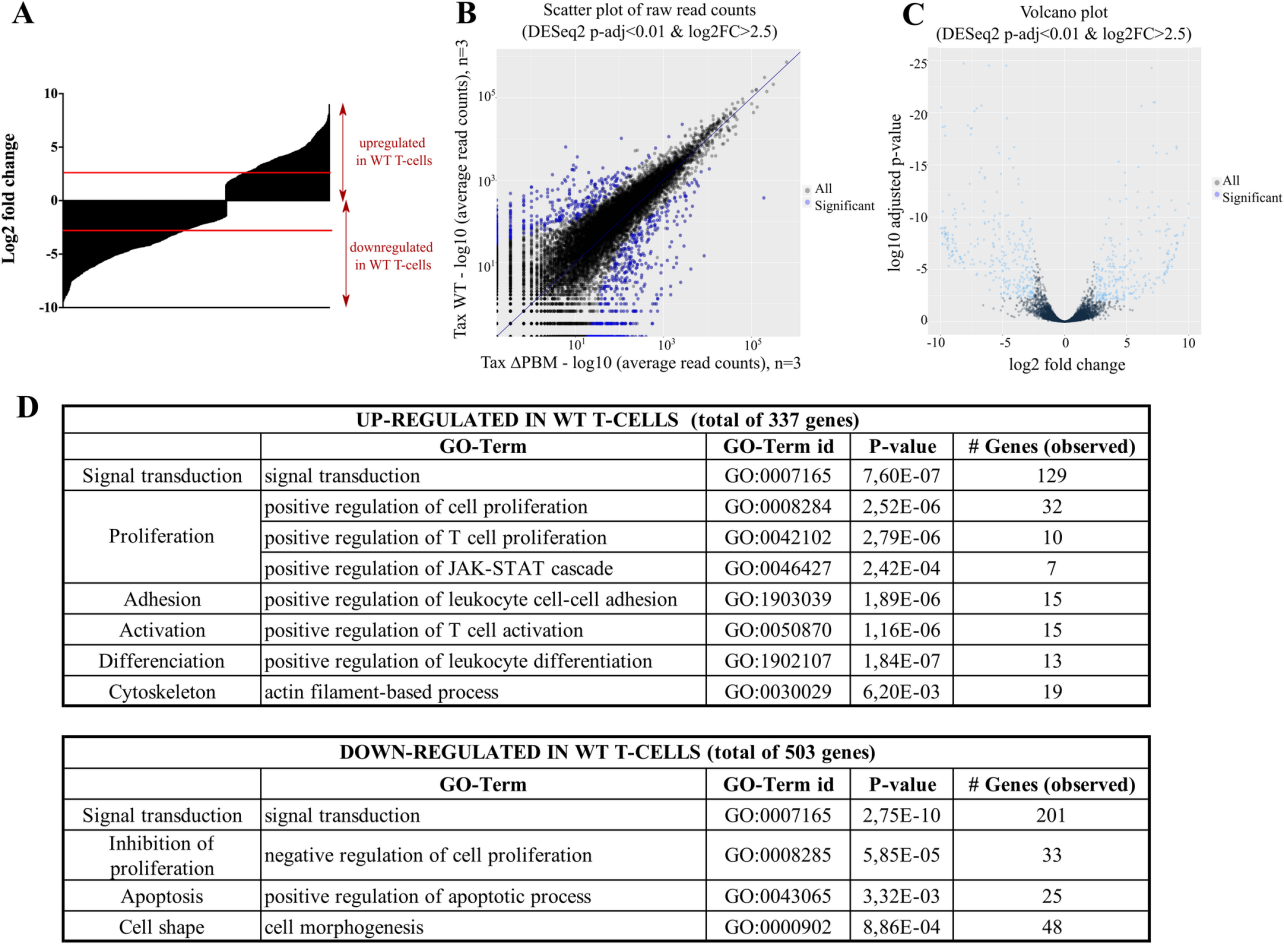
Genome-wide expression patterns of WT and ΔPBM T-cells by RNA-seq. (**A**) Graphic representation of transcript expression in WT T-cells compared to ΔPBM T-cells expressed as Log2 fold change. (**B**) Scatterplots comparing the per transcript read count values between WT and ΔPBM cells. The vast majority of differentially expressed transcripts (blue dots) in Δ*PBM* showed up-regulation while a smaller number of transcripts showed down-regulation. (**C**) Volcano plot comparing the per transcript fold change versus adjusted *P*-value. (**D**) Differential expression of transcripts (adjusted *P*-value < 0.01) by GO annotation according to the biological process category, calculated using Genomatix GeneRanker tool.

As PDZ proteins have been directly linked to the control of processes such as cytoskeletal organization, cell polarity and signal transduction pathways, we next investigated global transcriptional pathways that might be dysregulated by the PBM-PDZ interaction [17]. This was achieved by analyzing the transcriptome of cytoplasmic mRNA levels of both WT- and ΔPBM T-cells by RNA-sequencing (RNA-seq), at 5 months *in vitro* culture. Differential gene expression analysis using the DESeq2 package (adjusted *P*-value <0.01) resulted in the identification of 629 transcripts downregulated in WT T-cells, 503 of them displaying a fold change of at least 5.6 (Log2 fold-change <-2.5), whereas 400 transcripts were found to be upregulated, 337 of them displaying a fold change of at least 5.6 (Log2 fold-change >2.5) compared to the ΔPBM T-cells (Fig 7A, 7B and 7C). We looked for the transcriptional expression of genes coding for the PDZ proteins known to be involved in T-cell homeostasis, such as Scribble, MAGI-1, MAGI-3 and DLG1. We did not observe a significant difference in their expression levels, indicating that expression of these PDZ proteins is PBM-independent. In addition, the number of HTLV-1-related reads was identical under both conditions suggesting that the viral expression was not impaired in ΔPBM cells. Finally, we performed a gene ontology analysis of differentially expressed mRNAs (adjusted *P*-value <0.01; log2 fold-change of 2.5). Among the genes upregulated in WT cells we identified genes involved in cell proliferation such as *IL9* (fold change of 57) and cell activation such as *LCK* (fold change of 172) (Figs 7D and S6B). In contrast, genes downregulated in WT T-cells consisted of genes involved in inhibition of cell proliferation such as *CD9* (fold change of 129) and in apoptotic processes such as *RHOB* (fold change of 27). Furthermore, genes related to cytoskeleton organization were also identified as dependent on the Tax/PDZ interactions, some of them upregulated such as *CDC42BPA* while others showed decreased expression such as *FLNB*. Interestingly, the expression of class I regulatory PIK3R6 and PIK3CD subunits was upregulated in WT cells (fold change of 32 and 5.7 respectively). These proteins are implicated in the activation of the Akt/mTOR pathway, involved in cell proliferation and survival. We also identified a gene involved in the non-canonical Wnt pathway (*WNT5B*; increased expression; fold change of 16.7) and two genes associated with the canonical Wnt pathway (*WNT2* and *WNT1*; decreased expression; fold change of 16.7 and 147 respectively. This indicates that both canonical and non-canonical Wnt pathways might be modified by the interaction of Tax with PDZ proteins. In conclusion, these results suggest that the Tax PBM is involved in the transcriptional regulation of multiple genes implicated in T-cell proliferation, in the inhibition of apoptosis, as well as in cell polarity, in cytoskeletal and in morphological changes. Taken together, these *in vivo* and *in vitro* findings underline that PBM/PDZ recognition may be required for sustaining HTLV-1 mediated T-cell proliferation, for inducing cell polarity and cytoskeletal modifications and for triggering the immortalization of T-cells in hu-mice.

## Discussion

The generation of hu-mouse models, capable of multi-lineage human hematopoiesis has paved the way for the *in vivo* study of infection by human specific pathogens. Several human viruses and among them lymphotropic viruses have been extensively used in these models [22, 23, 30]. Thus, hu-mice have been used to approach the pathogenic activity of HTLV-1 Tax in a more biological model than cultured cells [25–27, 31]. Here we have investigated the role played by the Tax PBM in the lymphoproliferation triggered by HTLV-1 infection of hu-mice. To achieve this objective, we validated a new procedure to infect these animals with cloned proviruses. Thus, hu-mice were infected either by WT virus or by ΔPBM virus carrying a PBM-deleted genome, both produced after transfection of 293T cells with the corresponding provirus.

After examining the response of hu-mice to infection by WT or ΔPBM HTLV-1, we have characterized T-cells either freshly isolated from the spleen of these infected hu-mice or *in vitro* cultured. We observed an increased number and frequency of activated CD4+CD25+ T-cells in the peripheral blood of WT HTLV-1 hu-mice, albeit at a lower level in ΔPBM HTLV-1 infected mice. Splenomegaly, which was observed in both infected hu-mice, is mainly caused by a similar accumulation of CD3+ T-cells. But, once again, the number and frequency of CD4+CD25+ T-cells is significantly higher in WT than in ΔPBM hu-mice. Proviral loads and clonality in both types were similar, indicating that the PBM does not impact any of these parameters. Furthermore, we report that T-cell proliferation was severely impaired in hu-mice infected with the ACH-M22 provirus that carries the PBM, but unable to activate the NF-κB pathway. These data indicate for the first time that *in vivo* the PBM alone is unable to induce HTLV-1-mediated proliferation, but is only able to sustain the NF-κB-mediated proliferation. We next analyzed the proliferation of cells collected from the spleen of infected hu-mice and *in vitro* cultivated in presence of IL2. Short-term assays underlined that WT T-cells constantly proliferated over at least more than one year and were therefore considered as immortalized. In contrast, ΔPBM T-cells showed a restrained growth and experienced several death crisis suggesting that they were not immortalized. These data provided from infected hu-mice underline that the Tax-PBM is enhancing HTLV-1-mediated T-cell proliferation and is required for T-cell immortalization. A previous study has reported that the Tax PBM significantly increased HTLV-1-induced T-cell proliferation after *in vitro* cocultivation of human PBMCs (Peripheral Blood Mononuclear Cells) with irradiated cell lines producing WT or ΔPBM HTLV-1 [21]. These authors report that Tax PBM promotes HTLV-1-induced proliferation of human PBMCs, but that it is not required for virus-mediated immortalization of these cells, as they did not detect any difference in immortalization potential between WT Tax and ΔPBM Tax. As this conclusion concerning the implication of the Tax PBM in immortalization is differing from ours, it is tempting to speculate that such a difference may be related to the specific experimental approach of each study.

Previous studies have shown that the Tax PBM induced the mislocalization of Scribble [14, 15]. However, most if not all of the reported observations have been obtained through the use of transfected cell lines or HTLV-1 T-cell lines. Our study was performed with T-cells from hu-mice clearly indicates that the PBM/PDZ interactions are involved in the distribution pattern of Tax. Among the PDZ proteins, we focused our attention on the Scribble protein known to interact with Tax and to be involved in cell polarity, in T-cell development and proliferation. Based on the results of IF and PLA assays, a comparative analysis of *ex vivo* (immediately after their collection from the spleen) and *in vitro* (cultured in the presence of IL2) WT and ΔPBM T-cells underline that the Tax PBM plays a prominent role in the re-localization of both proteins. In WT cells, both Tax and Scribble were observed in large aggregates, mainly at the cell membrane. In contrast, such aggregates were not detected in ΔPBM cells, confirming the ability of Tax PBM to sequester Scribble.

The mislocalization of Scribble upon binding to the Tax PBM may be linked to morphological changes that differentially affect the actin cytoskeleton and the polarity of these WT and ΔPBM cells. In addition, the binding of the Tax PBM to Scribble may be responsible for the sustained proliferation of WT infected T-cells by negatively interfering with the tumor suppressor property of that PDZ protein. In contrast, the lack of interaction of Tax with Scribble may result in the decreased proliferation of ΔPBM T-cells. Further studies in hu-mice will aim at characterizing other PDZ proteins that interact with the Tax PBM, such as DLG-1, to unravel the possible link between these interactions and T-cell proliferation. It will also be of interest to test whether the acetylation of Tax lysine K10 (amino acid 346) located immediately upstream of the PBM could have an impact on the PBM/PDZ interactions, and finally on the localization of Tax and its function in T-cell proliferation.

It has been demonstrated that overexpression of Scribble attenuated NFAT reporter activity in anti-CD3/anti-CD28-stimulated Jurkat cells. By interacting with Scribble, Tax could counteract this negative effect on NFAT activation and thus stimulate T-cell proliferation [14]. Consequently Tax PBM association with PDZ proteins represents an essential event during the development and maintenance of the lymphoproliferative process. Moreover, this association appears to be linked to the exclusive HTLV-1-induced genetic instability observed in human PBMCs infected with WT-HTLV-1 [21]. In that context, to further explore the differences between cells expressing either Tax WT or Tax ΔPBM, we performed a comparative transcriptomic analysis that enabled the identification of a set of genes that are differentially expressed in either type of cells. Genes coding for PDZ proteins normally expressed in T lymphocytes such as Scribble, MAGI-I, MAGI-3 and DLG1 were found to be similarly expressed in WT and ΔPBM T-cells. In contrast, we noticed the deregulated transcription of genes involved in T-cell signaling and proliferation, in apoptosis induction and in cytoskeleton organization. The expression of PIK3 subunits retained our attention as these kinases activate Akt [32, 33]. We observed that the expression of PIK3 subunits is significantly higher in WT cells than in ΔPBM cells, suggesting a direct effect on Akt activation. It has been reported that Tax by binding to the PDZ DLG-1 protein counteracts the negative effects of the PTEN and PHLPP phosphatases and thus activates Akt [16]. Further studies will be needed to determine whether Scribble by interacting with Tax will have such an effect on Akt activation.

Finally, results from the genome wide transcriptome analysis, by supporting our *in vivo* and *in vitro* observations, propose that the Tax PBM plays an eminent role in the pathogenicity of HTLV-1. Further work is needed to establish that such an activity is dependent on PBM-PDZ interactions and to precisely determine which PDZ protein deregulates which set of genes.

As stated in the Introduction, the presence of the PBM was identified not only in Tax but also in several viral oncoproteins such as in the protein E4-ORF1 of adenovirus type 9 and in E6 proteins of several human papilloma viruses [7, 34]. It is worth noting that the presence of a PBM is linked to the ability of HPV-16 and HPV-18 to induce malignant tumors and that such a motif is absent in HPV subtypes that induce benign tumors (for example HPV-9 or-11) [35]. Our data converge to a similar conclusion and stress that the PBM/PDZ recognition is perturbing the regulation of processes such as cytoskeletal organization, cell polarity, cell proliferation. Consequently, the PBM represents a Tax domain endowed with an auxiliary activity essential in the induction and the maintenance of the HTLV-1-induced T-cell proliferation leading to a malignant proliferation. Thus targeting the PBM/PDZ nodes by small peptides is offering a novel strategy to slowdown the T-cell proliferation in HTLV-1 infected hu-mice [36].

## Materials and methods

### Cells and plasmids

The human embryonic kidney (HEK) 293T cells (American Type Culture Collection CRL-3216) were grown in Dulbecco’s modified Eagle’s medium (DMEM) supplemented with 10% fetal calf serum (FCS) (Sigma-Aldrich, France), 50 μg/ml streptomycin, 100 U/ml penicillin (Invitrogen, France).

The ACH plasmid is an infectious molecular clone of HTLV-1 [37]. The HTLV-1 provirus deleted from the PBM of Tax (ACH-ΔPBM) was constructed by introducing a TAA stop codon instead of the GAA codon in the Tax C-terminus resulting in loss of the last four amino-acids of Tax (ETEV: consensus PBM). This mutation was confirmed by DNA sequencing. The ACH-M22 plasmid encoding for a mutated Tax protein that do not activate the NF-κB pathway is a kind gift of Dr. F. Bex (Belgium).

### Cell transfection and Gag p19 ELISA

293T cells were plated at 5x10^5^ cells in a 6-well plate the day prior to transfection. Plasmid DNA (3.3 μg) was applied to the cells as calcium phosphate coprecipitates. Medium was changed 6h after transfection. One day later, supernatants were collected and analyzed for HTLV p19 antigen content by using the Retrotek ELISA-kit (ZeptoMetrix Corp., USA).

### Ethics statement

Anonymized human umbilical cord samples from the Maternity Ward of Hôpital Femme-Mère-Enfant (Bron, France) were obtained from healthy full-term newborns with written parental informed consent according to the guidelines of the medical and ethical committees of Hospices Civils de Lyon and of Agence de la Biomédecine, Paris, France. Experiments using cord blood were approved by both committees and were performed in full compliance with French law. Animal experimentation was performed in strict accordance with the French “Comité National de Réflexion Ethique sur l’Expérimentation Animale, n°15” and the ethical guidelines for the care of these mice of the Plateau de Biologie Expérimentale de la Souris (PBES, UMS 3444) at Ecole Normale Supérieure (ENS) de Lyon. This protocol has been approved by the Committee on the Ethics of Animal Experiments of ENS de Lyon (approval number: ENS_2014_043). All efforts were made to minimize animal suffering.

### Isolation of human CD34^+^ cells from cord blood samples

After density gradient centrifugation of human cord blood, CD34^+^ cells were enriched twice using immunomagnetic beads according to the manufacturer instructions (CD34^+^ MicroBead Kit, Miltenyi Biotec, Bergisch-Gladbach, Germany). Purity (≥ 95%) was evaluated by FACS analysis using human PE-CD34 antibody (Miltenyi Biotec). Cells were frozen before the transplantion when newborn mice were available.

### Generation and infection of humanized mice

NOD.Cg-Prkdc^scid^ Il2rγ^tm1Wjl^ Tg(HLA.A2.1)1Enge/SzJ (NSG-HLA-A2/HDD) were obtained from the Jackson Laboratory (Bar Harbor, USA) and bred and maintained under pathogen-free conditions at the PBES. Newborn males and females NSG mice (2 to 5 days old) were sub-lethally irradiated with 1.1 Gray (320 kV, 12.5 mA) from a X-ray irradiator (XRad-320, PXI Precision XRay, France) and intra-hepatically injected with 2x10^5^ human CD34^+^ hematopoietic stem cells isolated from cord blood samples [38]. After 10 weeks, humanized mice (≥ 30% hu-CD45+ cells in peripheral blood) were infected by HTLV-1. Infection and mice monitoring were performed in a Biosafety Level 3 Laboratory in accordance with the PBES guidelines. Lethally irradiated 293T cells (50 Gray, 320kV, 12.5 mA) transfected with full length or truncated HTLV-1 molecular clone were intra-peritoneally injected: the amount of irradiated cells to inject per mouse corresponds to the number of cells producing 70 ng of p19 in 24h-culture. Mock infected mice were injected with the same amount of irradiated non-transfected 293T. Hu-mice were daily monitored for signs of obvious suffering, such as weight loss, back arches and prostrated behavior. Peripheral blood was collected from the retro-orbital venous sinus under Isoflurane anesthesia. When mice were either suffering or displaying more than 10% circulating CD25+ T-cells, they were sacrificed after anesthesia. Tissue specimens (spleen, mesenteric lymph node and tibia bone) were collected and gently minced in PBS to obtain a single-cell suspension and immediately frozen in FCS containing 10% DMSO and kept at -80°C.

### Cell preparation and flow cytometry analysis

Spleens from infected hu-mice were harvested and analyzed at indicated time points following infection. To obtain a single-cell suspension, spleens were minced and passed through a nylon mesh. Red cell lysis was performed in red cell lysis buffer (Sigma, France) for 10 min. Cells were then washed and enumerated. For flow cytometry, single-cell suspensions were stained with the appropriate monoclonal antibody (S1 Table) or the respective isotype control antibody for 30 min in the dark at 4°C. Human lymphocytes first gated as hCD45+ cells were then defined as CD3+ T-cells containing the following subsets: DN, CD4-CD8-; DP, CD4+CD8+; and SP, CD4+ CD8- or CD4-CD8+. Absolute numbers of cells were determined by multiplying the number of nucleated cells by the percentage of positive cells for the indicated cell surface marker(s). For CADM-1 expression analysis, cells were stained with the primary antibody (Rabbit polyclonal antibody H-300 Santa Cruz Biotechnology, USA) for 2h at 4°C in PBS containing 5% FCS then washed twice and incubated 30 min at 4°C in the dark with a secondary fluorescent antibody anti-rabbit (A-11008 Molecular Probes, France). After two wash steps in FACS-buffer, fluorescence was measured on a flow cytometer (FACSCanto II, BD, San Jose, CA, USA). Cells were always gated to exclude doublet. Compensations were realized using Miltenyi MACS Comp Beads. Data were evaluated with BD Diva software (Becton Dickinson Immunocytometry Systems, Mountain View, CA) and analyzed using FlowJo software (Treestar, Ashland, USA). Results are expressed as the mean of % positivity of surface expression ± SEM or as the Mean Fluorescence Intensity (MFI).

To obtain cell lines derived from infected hu-mice, single cell suspensions were cultured in complete RPMI 1640 medium supplemented with 10% FCS (Sigma-Aldrich, France). Recombinant IL2 (20 U/ml) (Peprotech, France) was added to the cultures every 3 days. After one month of culture, selected cell lines were tested for the relevant proviral sequence and weekly monitored for their proliferation.

### Histology and immunohistochemistry

Spleen samples were fixed with 4% paraformaldehyde in PBS, embedded in paraffin, sectioned and stained with H&E solution. An indirect immunoperoxidase technique with commercially available monoclonal antibodies to CD3 and rabbit polyclonal antibody to Tax (kind gift of Dr. B. Cullen) was applied to the tissue sections as previously described [27]. Pictures were analyzed with ImageJ software.

### Immunofluorescent (IF) staining and proximity ligation assay (PLA)

Slides or ibiTreat μ-dishes (IBIDI) were pre-coated with poly-L-lysine (Sigma-Aldrich, France) 20 μg/ml for IF and 10 μg/ml for PLA. To detect Tax and Scribble proteins, T-cells isolated from spleens were added to the slides before being fixed with 3% paraformaldehyde in PBS for 10 min at room temperature. Cells were then permeabilized with TritonX-100 or methanol at -20°C. For IF staining, cells were blocked with 5% FCS then stained with primary and secondary antibodies and mounting medium with DAPI (Duolink, Sigma, France). Polyclonal goat antibodies to Scribble (C-20; Santa Cruz Biotechnology), rabbit antibodies to Tax (kind gift of Dr. B. Cullen) and appropriate controls were used. Phalloidin, fluorescein isothyocyanate labeled (Sigma-Aldrich) interacts with polymeric actin. PLA was carried out with Duolink In Situ-Fluorescence Red kit (Sigma-Aldrich) according to the manufacturer’s instructions. Negative controls were performed on T-cells in the absence of antibodies to Scribble. Microscopic examination was performed using a Zeiss LSM710 confocal microscope (Carl Zeiss Jena, inc, Germany) or an Axioimager Z1 epifluorescence microscope (Carl Zeiss Jena, inc, Germany). Images were analyzed using ImageJ software.

### Statistical analysis

Statistical tests were performed using GraphPad PRISM software. When n≤ 5 in one or both groups, they are one-tailed and the non-parametric Wilcoxon-Mann-Whitney test was performed. When n> 5 in one or both groups, the parametric Student t-test was performed if variance are equal (F-test with *P*-value > 0.05). If not (F-test with *P*-value < 0.05), the non-parametric Wilcoxon-Mann-Whitney *U* test was performed. Statistical analysis of hu-CD3 subpopulations was performed with one-way ANOVA test. The results were considered statistically significant when *P*-value < 0.05.

### Western blot analysis

Cells were lysed with sample buffer (10 mM HEPES, pH 7.9, 500 mM NaCl, 3 mM MgCl2, 1 mM DTT, 1 mM PMSF, and 0.5% Triton X-100 supplemented with protease inhibitors). After incubation on ice for 60 min, whole cell lysates were centrifuged at 15,000 g for 10 min at 4°C to remove the debris. Protein concentration of the cleared lysates was determined using the Bradford assay. Cell lysates (15μg) were size-separated by electrophoresis on a 12% SDS-polyacrylamide gel (3h migration at 20 mA) and transferred onto PVDF membranes. The blot was blocked in PBS-5% milk and incubated with anti-Tax antibodies (1:1,000; kind gift of B. Cullen), anti-β-actin (1:5,000) obtained from Sigma-Aldrich (clone AC-15). After several washes, horseradish peroxidase (HRP)-conjugated anti-rabbit IgG (1:10,000; Cell Signaling, The Netherlands) or HRP-linked anti-mouse IgG (1:10,000; GE Healthcare, France) were added to the membranes which were washed again several times and subsequently incubated with the Western Lightning ECL solution (Thermo Fisher Scientific, France). Images were captured using a ChemiDoc Imaging system (Biorad, France).

### DNA and RNA extraction, quantitative real-time PCR (qPCR) and proviral load

Genomic DNA was extracted from the single cell suspension using the Nucleospin Blood kit (Macherey-Nagel, Düren, Germany) according to the manufacturer’s instructions. PVL was measured by qPCR with HTLV-1 *tax*-specific primers. The PVL was calculated as previously described [27] and expressed as the number of *pX* copies per 100 human cells.

RNA was extracted from the single cell suspension using RNAzol RT (Sigma Aldrich, France) according to the manufacturer’s instructions, and resuspended in 10 μl of RNAse-free water and treated with 10 U of RNase-free DNase I (Qiagen, Hilden, Germany) for 15 min at 30°C and then for 15 min at 60°C. 500 ng of total RNA were then retro-transcribed at 42°C during 50 min in a total volume of 20 μl reaction buffer containing 100 U of SuperScript II reverse transcriptase (RT; Invitrogen, CA, USA). A reaction without RT was performed as a control for genomic DNA contamination. The mRNA levels were normalized using 3 different housekeeping genes *(ACTB*, *RSP11* and *RSP14*) chosen to be the most stable in our model with BestKeeper and NormFinder algorithms. Quantitative real-time PCR (qPCR) was performed using the FastStart Universal SYBR Green Master (Roche, Mannheim, Germany) on a StepOnePlus system (Applied Biosystem, CA, USA). The initial denaturation step at 95°C for 10 min was followed by 40 cycles with one cycle consisting of 10s at 95°C, 30s at 60°C, and 15s at 72°C.

### Primers

The nucleotide sequences of the primers were used for RT-PCR, proviral load measurement and DNA sequencing of the mutation of *Tax* gene are shown in S2 Table.

### High-throughput sequencing (HTS) of HTLV-1 integration sites

To determine the number and abundance of HTLV-1 infected clones in humanized mice, we used an improved quantitative HTS method to map the proviral integration sites in the human genome [39]. Libraries were prepared starting from 500 ng DNA and sequenced on an Illumina MiSeq instrument. 150 bp paired-end reads were acquired and sequencing reads that supported either the 5’ or the 3’ LTR-host junctions were retained. The number of unique integration sites (UIS) was determined as previously described [40].

### Cell lysis and RNA-seq analysis

T lymphocytes isolated from the spleen of WT or ΔPBM infected hu-mice were cultured in complete RPMI medium containing IL2. Cells (5x10^6^) were then washed with ice-cold PBS, centrifuged at 500 g for 5 min at 4°C and lysed in 1 ml of lysis buffer (10mM Tris-HCl pH7.5, 5mM MgCl2, 100mM KCl, 2mM DTT, protease inhibitor EDTA-free (Roche, Mannheim, Germany), 1% Triton X-100). Lysates were gently homogenized and incubated at 4°C for 10 min, centrifuged at 1,300 *g* for 10 min at 4°C and the supernatant was recovered. Total RNA was extracted from cellular extracts using Trizol and subjected to cDNA library construction using the smartseq2 protocol [41].

### Mapping of high-throughput sequencing reads

Reads were first split with respect to their 5′-barcode sequence. After this, 5′-barcode and 3′-adaptor sequences were removed from reads. Reads were then aligned to a custom set of sequences corresponding to ribosomal RNA and tRNA sequences using Bowtie [42] in order to remove contaminants. Remaining reads were aligned to the human genome and transcriptome (hg19 assembly) using TopHat2 [43].

### Transcript-level quantification and differential gene expression

The alignment files obtained from TopHat2 were used to count reads mapping to the 5’UTR coding sequence and 3’UTR of human transcripts using HTSeq [44] and the UCSC hg19 gene annotation file. Differential gene expression was performed using the R package DESeq2 [45].

### Data analysis for HTS

Only genes with an adjusted *P*-value ≤ 0.01 and a log2 foldchange superior to 2.5 were selected. GeneOntology was done using the GeneRanker tool of Genomatix software.

## Supporting information

S1 Table Monoclonal antibodies and isotype controls used in flow cytometry. IgG indicates immunoglobulin G; FITC, fluorescein isothiocyanate; PE, phycoerythrin; APC, allophycocyanin; V450, BD Horizon *V450*, a coumarin dye excited by the violet laser.
(DOCX)

S2 Table Primers used for PCR and RT-PCR. (DOCX)

S3 Table Human T-cell subsets in the peripheral blood (PB) of infected hu-mice, seven weeks after HTLV-1 infection. ^a^hu-mice were intraperitoneally inoculated with 293T cells transfected with ACH-WT (n = 5), ACH-ΔPBM (n = 8), ACH-M22 (n = 3) or mock infected (n = 3) and then X-irradiated. Peripheral blood samples were collected at 7 weeks after infection.
^b^Frequencies of the CD3+, CD4+CD25+, and CD8+CD25+ cells in the peripheral blood were calculated out of hu-CD45+ cells.
(DOCX)

S4 Table Proviral load, Tax mRNA expression and pathological features in hu-mice infected with HTLV-1. Hu-mice were intraperitoneally inoculated with irradiated 293T cells transfected with the indicated plasmids. They were sacrificed at indicated times. Proviral load is expressed as number of proviral copies per 10^5^ splenocytes. LN = lymph node; nd = not determined. Levels of Tax mRNA in splenocytes isolated from HTLV-1-infected hu-mice were measured by RT-qPCR as indicates in Fig 2B.
(DOCX)

S5 Table Sequencing analysis of representative samples. ^a^DNA from ACH plasmids, from mouse splenocytes and from cultured T-cells were extracted as indicated in Materials and methods, subjected to PCR amplification and sequenced by using the primers listed in S1 Table.
^b^Nucleotide sequence of Tax in italic and of PBM in bold. Note the mutation of **GAA** into **TAA** (stop codon) in ACH ΔPBM plasmid, in the spleen of ΔPBM infected hu-mice and in ΔPBM T-cells cultured *in vitro*.
(DOCX)

S6 Table Human T-Cell subsets in the bone marrow (BM), lymph nodes (LN) and the spleen (SPL) of infected hu-mice at the autopsy. ^a^hu-mice were intraperitoneally inoculated with 293T cells transfected with ACH-WT (n = 13), ACH-ΔPBM (n = 13), ACH-M22 (n = 3) or mock infected (n = 3) and then X-irradiated.
^b^Bone Marrow (BM) from tibia, Spleen (SPL) and mesenteric lymph nodes (LN) were collected and analyzed by FACS for indicated surface markers.
^c^Frequency of the CD3+, CD4+CD25+, and CD8+CD25+ cells were calculated out of the number of hu-CD45+ cells.
(DOCX)

S1 Fig Tax transcriptional activation of CREB/ATF- and NF-κB-dependent reporter genes. 293T cells (9 × 10^4^/ 24 well) were cotransfected with the indicated ACH plasmid (100 ng), TK-*Renilla* (5 ng) reporter plasmid together with the HTLV-1 LTR-luc (A), or the κB-luc (B) as calcium phosphate coprecipitates. Cell lysates were harvested 48h after transfection and luciferase activity was determined using the Dual Luciferase Assay System (Promega). The histogram presents the average fold activation over control values for 2 independent experiments in triplicate; data are presented as mean ± SEM.
(TIF)

S2 Fig Number of unique integration sites (UIS) in both types of infected hu-mice. The number of independent HTLV-1-infected clones was determined by HTS clonality analysis in splenocytes (8 WT and 9 ΔPBM). Bar represents mean. Student *t*-test, *P* = 0.3021.
(TIF)

S3 Fig Immunohistochemistry of spleen sections of different WT and ΔPBM infected hu-mice. Staining with anti-Tax antibodies revealed an infiltration of T-lymphocytes with a nuclear localization of Tax.
(TIF)

S4 Fig FACS analysis of splenic T-cells in HTLV-1 infected hu-mice. Splenocytes from WT or ΔPBM-infected hu-mice were harvested 7 weeks after infection. Representative profile for CD4, CD8, and CD25 expression on gated hu-CD3+ cells.
(TIF)

S5 Fig (A) **Size (FSC for Forward Scatter) and (B) Granularity (SSC for Side Scatter**) of CD4+CD25+ T-cells in the spleen of WT and **Δ**PBM hu-mice.
(TIF)

S6 Fig Gene Ontology Analysis. (A) Reads were mapped on the human genome (hg19). They are specific of gene exons and do not map on repeated sequences. Shown is the number of reads in the WT cells (in purple) and **Δ**PBM cells (in orange). (B) Detailed list of the differential expression of transcripts (adjusted *P*-value < 0.01) by GO annotation according to the biological process category, calculated using Genomatix GeneRanker tool.
(TIF)
