# Supplementary material for: PDZ domain-binding motif of Tax sustains T-cell proliferation in HTLV-1-infected humanized mice
Source: PLoS Pathog. 2018 Mar 22;14(3):e1006933. doi: 10.1371/journal.ppat.1006933 (PMC5882172; doi:10.1371/journal.ppat.1006933)
Supplement: S1 Table — IgG indicates immunoglobulin G; FITC, fluorescein isothiocyanate; PE, phycoerythrin; APC, allophycocyanin; V450, BD Horizon V450, a coumarin dye excited by the violet laser. (DOCX) [file ppat.1006933.s001.docx]

| **Antigen** | **Conjugate** | **Isotype** | **Clone** | **Origin** | **Company** |
| --- | --- | --- | --- | --- | --- |
| hCD45 | V450 | IgG1 | HI30 | Mouse | BD Biosciences |
| hCD3 | FITC | IgG2a | HIT3a | Mouse | BD Biosciences |
| hCD8 | PE | IgG1 | RPA-T8 | Mouse | BD Biosciences |
| hCD4 | PE-Cy7 | IgG1 | SK3 | Mouse | BD Biosciences |
| hCD25 | APC | IgG1 | M-A251 | Mouse | BD Biosciences |
| hCD34 | PE | IgG1 | 581 | Mouse | BD Biosciences |
| IgG1 isotype control | V450 | IgG1 | MOPC-21 | Mouse | BD Biosciences |
| IgG2a isotype control | FITC | IgG2a | G155-178 | Mouse | BD Biosciences |
| IgG1 isotype control | PE | IgG1 | MOPC-21 | Mouse | BD Biosciences |
| IgG1 isotype control | PE-Cy7 | IgG1 | MOPC-21 | Mouse | BD Biosciences |
| IgG1 isotype control | APC | IgG1 | MOPC-21 | Mouse | BD Biosciences |
