# Supplementary material for: PDZ domain-binding motif of Tax sustains T-cell proliferation in HTLV-1-infected humanized mice
Source: PLoS Pathog. 2018 Mar 22;14(3):e1006933. doi: 10.1371/journal.ppat.1006933 (PMC5882172; doi:10.1371/journal.ppat.1006933)
Supplement: S2 Table — (DOCX) [file ppat.1006933.s002.docx]

| **ID** | **Forward primer** | **Reverse primer** |
| --- | --- | --- |
| *ACTB* | 5’–TGAGCTGCGTGTGGCTCC–3’ | 5’–GGCATGGGGGAGGGCATACC–3’ |
| *RPS11* | 5’-TGCACTACATCCGCAAGTACA-3’ | 5’-CACGTTGAAGCGCACTGTCT-3’ |
| *RPS14* | 5’-CGTGTGACTGGTGGGATGAA-3’ | 5’-TCCAGGGGTCTTGGTCCTAT-3’ |
| genomic *tax* | 5′-GTTGTATGAGTGATTGGCGGGGTAA-3′ | 5′-TGTTTGGAGACTGTGTACAAGGCG-3′ |
| *TAX* | 5’-ATCCCGTGGAGACTCCTCAA-3’ | 5’-CCAAACACGTAGACTGGGTATCC-3’ |
| *PBM_TAX* first | 5'-TCACAAATTTCAAACCAAGGCC+-3' | 5'-AACATGGGGAGGAAATGGGT-3' |
| *PBM_TAX* internal | 5'-CTACTCTCACACGGCCTCAT-3' | 5'-GGACACGTCAGGGCCTAG-3' |
