# Supplementary material for: PDZ domain-binding motif of Tax sustains T-cell proliferation in HTLV-1-infected humanized mice
Source: PLoS Pathog. 2018 Mar 22;14(3):e1006933. doi: 10.1371/journal.ppat.1006933 (PMC5882172; doi:10.1371/journal.ppat.1006933)
Supplement: S3 Table — ahu-mice were intraperitoneally inoculated with 293T cells transfected with ACH-WT (n = 5), ACH-ΔPBM (n = 8), ACH-M22 (n = 3) or mock infected (n = 3) and then X-irradiated. Peripheral blood samples were collected at 7 weeks after infection. bFrequencies of the CD3+, CD4+CD25+, and CD8+CD25+ cells in the peripheral blood were calculated out of hu-CD45+ cells. (DOCX) [file ppat.1006933.s003.docx]

| ^a^Infection | #mouse | % hu-CD45 before infection | % hu-CD45  at 7 weeks | % CD3+ of hu-CD45 | % SP4 of hu-CD3 | % SP8 of hu-CD3 | % DP of hu-CD3 | % DN of hu-CD3 | ^b^ % CD4+ CD25+ of hu-CD45 | ^b^ % CD8+ CD25+ of hu-CD45 |
| --- | --- | --- | --- | --- | --- | --- | --- | --- | --- | --- |
|  |  |  |  |  |  |  |  |  |  |  |
| WT | #343 | 28.6 | 85.2 | 99.2 | 53.2 | 25.1 | 20.6 | 1.1 | 15.9 | 1.7 |
|  | #352 | 38.0 | 98.7 | 99.6 | 50.9 | 41.6 | 7.1 | 0.3 | 13.4 | 3.4 |
|  | #353 | 40.3 | 83.1 | 99.2 | 72.2 | 23.1 | 4.2 | 0.6 | 19.2 | 1.0 |
|  | #401 | 31.7 | 40.7 | 96.1 | 42.9 | 39.3 | 17.3 | 0.4 | 12.5 | 0.6 |
|  | #403 | 32.3 | 89.8 | 99.3 | 66.2 | 27.8 | 5.8 | 0.2 | 18.5 | 1.2 |
|  |  |  |  |  |  |  |  |  |  |  |
| ΔPBM | #334 | 21.8 | 96 | 99.0 | 44.7 | 39.2 | 14.3 | 1.7 | 9.8 | 2.7 |
|  | #338 | 32.8 | 36.8 | 56.3 | 66.9 | 28.5 | 2.6 | 1.9 | 2.8 | 0.3 |
|  | #344 | 33.0 | 88.1 | 98.8 | 58.3 | 32.3 | 9.0 | 0.4 | 13.3 | 2.5 |
|  | #347 | 26.7 | 51.6 | 87.7 | 77.3 | 20.8 | 1.4 | 0.4 | 16.3 | 1.8 |
|  | #349 | 27.6 | 96.3 | 99.6 | 34.6 | 41.5 | 22.5 | 1.4 | 10.1 | 1.3 |
|  | #399 | 48.2 | 97.4 | 96.8 | 51.1 | 30.6 | 17.0 | 1.4 | 5.3 | 1.3 |
|  | #406 | 63.3 | 96.3 | 98.5 | 67.1 | 26.8 | 5.6 | 0.4 | 11.0 | 0.7 |
|  | #455 | 37.4 | 90.4 | 96.3 | 65.1 | 29.3 | 5.3 | 0.3 | 8.3 | 1.4 |
|  |  |  |  |  |  |  |  |  |  |  |
| M22 | #402 | 33.0 | 28.4 | 26.5 | 78.6 | 20.0 | 1.4 | 0 | 0.8 | 0 |
|  | #409 | 51.9 | 36.0 | 49.6 | 65. 7 | 28.9 | 1.9 | 3.5 | 2.3 | 0.1 |
|  | #414 | 16.7 | 24.6 | 37.9 | 71.4 | 23.6 | 2.1 | 2.9 | 1.6 | 0 |
|  |  |  |  |  |  |  |  |  |  |  |
| Mock | #413 | 47.5 | 11.4 | 58.3 | 77.1 | 20.0 | 2.86 | 0 | 3.3 | 0 |
|  | #422 | 35.3 | 28.2 | 33.1 | 35.7 | 64.3 | 0 | 0 | 1.6 | 0 |
|  | #446 | 26.5 | 23.7 | 30.2 | 64.1 | 33.3 | 0.6 | 2.0 | 1.0 | 0 |
