# Supplementary material for: PDZ domain-binding motif of Tax sustains T-cell proliferation in HTLV-1-infected humanized mice
Source: PLoS Pathog. 2018 Mar 22;14(3):e1006933. doi: 10.1371/journal.ppat.1006933 (PMC5882172; doi:10.1371/journal.ppat.1006933)
Supplement: S4 Table — Hu-mice were intraperitoneally inoculated with irradiated 293T cells transfected with the indicated plasmids. They were sacrificed at indicated times. Proviral load is expressed as number of proviral copies per 105 splenocytes. LN = lymph node; nd = not determined. Levels of Tax mRNA in splenocytes isolated from HTLV-1-infected hu-mice were measured by RT-qPCR as indicates in Fig 2B. (DOCX) [file ppat.1006933.s004.docx]

| Infection | #mouse | Week of sacrifice | Pathological features | Spleen weight (mg) | # hCD45 (x10^6^) | Proviral load (%) | Tax mRNA expression |
| --- | --- | --- | --- | --- | --- | --- | --- |
| WT | #397 | 3 | « tumor-like » nodules in the spleen; splenomegaly | 225 | 60.3 | 2 | 0.05 |
|  | #418 | 4 | none | 118 | 24.9 | 13 | 0.35 |
|  | #448 | 4 | 1 enlarged LN; splenomegaly | 251 | 58.6 | 35 | 0.32 |
|  | #458 | 4 | splenomegaly | 415 | 99.1 | 20 | 0.28 |
|  | #313 | 5 | none | nd | nd | 38 | nd |
|  | #355 | 6 | 1 enlarged LN | 105 | 30.4 | 45 | 0.33 |
|  | #356 | 6 | 3 enlarged LN ; splenomegaly | 206 | 0 | 57 | 1.09 |
|  | #357 | 6 | none | 100 | 28.6 | 4 | 0.05 |
|  | #343 | 7 | 1 enlarged LN; splenomegaly | 250 | 55.7 | 80 | nd |
|  | #352 | 7 | 1 enlarged LN ; splenomegaly | 641 | 170 | 95 | 1.93 |
|  | #353 | 7 | splenomegaly | 310 | 80 | 21 | 0.34 |
|  | #401 | 7 | splenomegaly | 274 | 68.5 | 14 | 0.16 |
|  | #403 | 7 | none | 174 | 39.8 | 18 | 0.81 |
|  |  |  |  |  |  |  |  |
| ΔPBM | #340 | 4 | none | nd | 39.2 | 18 | 0.02 |
|  | #404 | 4 | splenomegaly | 214 | 55 | 26 | 1.87 |
|  | #407 | 4 | none | 183 | 48.9 | 26 | 0.7 |
|  | #420 | 4 | none | 37 | 10.2 | 20 | 1.64 |
|  | #339 | 6 | 1 enlarged LN; splenomegaly | 340 | 87.3 | 66 | 2.71 |
|  | #334 | 7 | splenomegaly | 212 | 55.8 | 23 | 0.78 |
|  | #338 | 7 | none | 71 | 19.8 | 2 | 0.42 |
|  | #344 | 7 | 1 enlarged LN ; splenomegaly | 420 | 99.1 | 23 | 1.42 |
|  | #347 | 7 | none | 179 | 46.8 | 17 | 0.23 |
|  | #349 | 7 | none | 167 | 38.9 | 22 | 4.08 |
|  | #399 | 7 | splenomegaly | 230 | 62.5 | 16 | 0.46 |
|  | #406 | 7 | splenomegaly | 390 | 102.6 | 16 | 0.3 |
|  | #455 | 7 | splenomegaly | 350 | 87.3 | 15 | 0.37 |
|  |  |  |  |  |  |  |  |
| M22 | #402 | 7 | none | 80 | 18.9 | <0.5 | nd |
|  | #409 | 7 | none | 160 | 13.5 | <0.5 | nd |
|  | #414 | 7 | none | 106 | 36.3 | <0.5 | nd |
|  |  |  |  |  |  |  |  |
| Mock | #413 | 7 | none | 80 | 18.9 | neg | nd |
|  | #422 | 7 | none | 135 | 35.2 | neg | nd |
|  | #446 | 7 | none | 61 | 14.8 | neg | nd |
