# Supplementary material for: PDZ domain-binding motif of Tax sustains T-cell proliferation in HTLV-1-infected humanized mice
Source: PLoS Pathog. 2018 Mar 22;14(3):e1006933. doi: 10.1371/journal.ppat.1006933 (PMC5882172; doi:10.1371/journal.ppat.1006933)
Supplement: S5 Table — aDNA from ACH plasmids, from mouse splenocytes and from cultured T-cells were extracted as indicated in Materials and methods, subjected to PCR amplification and sequenced by using the primers listed in S1 Table. bNucleotide sequence of Tax in italic and of PBM in bold. Note the mutation of GAA into TAA (stop codon) in ACH ΔPBM plasmid, in the spleen of ΔPBM infected hu-mice and in ΔPBM T-cells cultured in vitro. (DOCX) [file ppat.1006933.s005.docx]

| ^a^DNA | ^b^FORWARD | REVERSE |
| --- | --- | --- |
| ACH WT plasmid | *AAACATTTCCGC-***GAAACAGAAGTC-***TGAAAAGGTCAG* |  |
| ACH ∆PBM plasmid | *AAACATTTCCGA-***TAAACAGAAGTC-***TGAAAAGGTCAG* |  |
|  |  |  |
| WT mouse #352 | *AAACATTTCCGC-***GAAACAGAAGTC-***TGAAAAGGTCAG* | *CTGACCTTTTCA-***GACTTCTGTTTC-***GCGGAAATGTTT* |
| WT mouse #403 | *AAACATTTCCGC-***GAAACAGAAGTC-***TGAAAAGGTCAG* | *CTGACCTTTTCA-***GACTTCTGTTTC-***GCGGAAATGTTT* |
|  |  |  |
| ∆PBM mouse #339 | *AAACATTTCCGA*-**TAAACAGAAGTC**-*TGAAAAGGTCAG* | *CTGACCTTTTCA-***GACTTCTGTTTA-***TCGGAAATGTTT* |
| ∆PBM mouse #406 | *AAACATTTCCGA*-**TAAACAGAAGTC**-*TGAAAAGGTCAG* | *CTGACCTTTTCA-***GACTTCTGTTTA-***TCGGAAATGTTT* |
|  |  |  |
| WT T-cells | *AAACATTTCCGC-***GAAACAGAAGTC-***TGAAAAGGTCAG* | *CTGACCTTTTCA-***GACTTCTGTTTC-***GCGGAAATGTTT* |
| ∆PBM T-cells | *AAACATTTCCGA*-**TAAACAGAAGTC**-*TGAAAAGGTCAG* | *CTGACCTTTTCA-***GACTTCTGTTTA-***TCGGAAATGTTT* |
