# Supplementary material for: PDZ domain-binding motif of Tax sustains T-cell proliferation in HTLV-1-infected humanized mice
Source: PLoS Pathog. 2018 Mar 22;14(3):e1006933. doi: 10.1371/journal.ppat.1006933 (PMC5882172; doi:10.1371/journal.ppat.1006933)
Supplement: S6 Table — ahu-mice were intraperitoneally inoculated with 293T cells transfected with ACH-WT (n = 13), ACH-ΔPBM (n = 13), ACH-M22 (n = 3) or mock infected (n = 3) and then X-irradiated. bBone Marrow (BM) from tibia, Spleen (SPL) and mesenteric lymph nodes (LN) were collected and analyzed by FACS for indicated surface markers. cFrequency of the CD3+, CD4+CD25+, and CD8+CD25+ cells were calculated out of the number of hu-CD45+ cells. (DOCX) [file ppat.1006933.s006.docx]

| ^a^Infection | #mouse | ^b^organ | % hu-CD45 | % CD3+ of hu-CD45 | % SP4 of hu-CD3 | % SP8 of hu-CD3 | % DP of hu-CD3 | % DN of hu-CD3 | ^c^% CD4+ CD25+  of hu-CD45 | ^c^% CD8+ CD25+ of hu-CD45 |
| --- | --- | --- | --- | --- | --- | --- | --- | --- | --- | --- |
|  |  |  |  |  |  |  |  |  |  |  |
| WT | #313 | BM | 77.3 | 92.5 | 40.4 | 46.9 | 12.0 | 0.7 | 16.4 | 8.7 |
|  |  | SPL | 46.0 | 87.6 | 38.0 | 44.7 | 2.4 | 15.0 | 14.3 | 2.3 |
|  | #343 | BM | 71.7 | 61.7 | 66.7 | 23.7 | 6.4 | 3.2 | 19.8 | 1.6 |
|  |  | LN | 89.5 | 86.3 | 54.4 | 29.4 | 14.7 | 1.5 | 28.0 | 4.8 |
|  |  | SPL | 59.2 | 96.4 | 54.9 | 32.2 | 6.0 | 7.0 | 26.5 | 4.4 |
|  | #352 | BM | 19.3 | 93.3 | 38.9 | 44.4 | 16.7 | 0.1 | 33.4 | 14.2 |
|  |  | LN | 89.2 | 88.5 | 47.2 | 44.3 | 7.3 | 1.2 | 21.6 | 7.4 |
|  |  | SPL | 66.5 | 95.5 | 30.5 | 39.1 | 0.8 | 29.6 | 17.5 | 6.9 |
|  | #353 | BM | 40.9 | 91.0 | 70.1 | 22.6 | 7.0 | 0.4 | 46.1 | 2.0 |
|  |  | SPL | 73.8 | 97.9 | 70.5 | 24.5 | 2.2 | 2.8 | 32.3 | 2.3 |
|  | #355 | BM | 73.7 | 98.1 | 77.4 | 12.3 | 9.8 | 0.5 | 56.2 | 3.8 |
|  |  | LN | 98 | 86.8 | 55.3 | 33.4 | 10.1 | 1.2 | 31.2 | 9.2 |
|  |  | SPL | 57.5 | 95.4 | 27.3 | 26.6 | 1.1 | 45.0 | 15.9 | 23.7 |
|  | #356 | BM | 24.6 | 98.8 | 53.7 | 39.0 | 4.9 | 2.4 | 35.0 | 8.4 |
|  |  | LN | 92.2 | 96.7 | 58.4 | 29.2 | 10.6 | 1.8 | 47.8 | 2.0 |
|  |  | SPL | 20.2 | 98.8 | 47.9 | 40.3 | 7.5 | 4.3 | 30.2 | 6.2 |
|  | #357 | BM | ND |  |  |  |  |  |  |  |
|  |  | LN | 99.4 | 66.6 | 77.8 | 17.8 | 2.3 | 2.0 | 6.6 | 2.3 |
|  |  | SPL | 46.7 | 88.1 | 86.5 | 9.7 | 1.1 | 2.6 | 11.4 | 0.8 |
|  | #397 | BM | 93.1 | 79.2 | 40.7 | 10.2 | 16.9 | 32.2 | 6.7 | 43.0 |
|  |  | SPL | 72.7 | 85.2 | 72.5 | 21.1 | 5.6 | 0.8 | 25.5 | 0.8 |
|  | #401 | BM | 29.1 | 98.7 | 48.3 | 11.0 | 10.1 | 30.7 | 10.3 | 1.1 |
|  |  | SPL | 31.8 | 70.9 | 40.0 | 25.0 | 7.3 | 27.7 | 8.3 | 1.2 |
|  | #403 | BM | 88.4 | 98.3 | 65.3 | 29.4 | 5.1 | 0.2 | 16.3 | 1.8 |
|  |  | SPL | 66.5 | 95.9 | 60.7 | 33.3 | 2.1 | 3.9 | 13.0 | 1.1 |
|  | #418 | BM | 92.1 | 90.0 | 42.9 | 41.4 | 6.2 | 9.6 | 9.5 | 2.4 |
|  |  | LN | 94.2 | 76.1 | 40.8 | 50.9 | 2.2 | 6.1 | 4.1 | 4.6 |
|  |  | SPL | 82.2 | 88.7 | 52.4 | 35.3 | 5.3 | 7.1 | 12.0 | 0 |
|  | #448 | BM | 94.4 | 90.8 | 47.4 | 29.6 | 3.8 | 19.2 | 22.5 | 2.1 |
|  |  | LN | 88.1 | 80.4 | 22.3 | 42.1 | 4.1 | 31.6 | 19.3 | 8.1 |
|  |  | SPL | 87.2 | 94.3 | 39.5 | 32.7 | 1.8 | 26.0 | 30.3 | 0.2 |
|  | #458 | BM | 53.7 | 70.3 | 45.5 | 29.7 | 5.7 | 19.1 | 11.2 | 1.7 |
|  |  | SPL | 56.8 | 92.4 | 66.7 | 11.1 | 14.0 | 8.3 | 12.0 | 0.4 |
|  |  |  |  |  |  |  |  |  |  |  |
| ΔPBM | #334 | BM | 64.7 | 89.5 | 53.0 | 31.3 | 13.7 | 2.0 | 24.2 | 5.1 |
|  |  | SPL | 77.5 | 97.4 | 33.0 | 42.9 | 3.5 | 20.6 | 13.7 | 3.6 |
|  | #338 | BM | 16.1 | 12.3 | 68.2 | 24.2 | 6.0 | 1.7 | 2.0 | 0.1 |
|  |  | LN | 95.8 | 47.0 | 50.6 | 44.3 | 2.8 | 2.4 | 2.2 | 0.6 |
|  |  | SPL | 73 | 22.3 | 57.1 | 35.1 | 5.1 | 2.7 | 2.4 | 0.3 |
|  | #339 | BM | 44.5 | 98.7 | 48.4 | 30.9 | 20.6 | 0.1 | 34.6 | 9.3 |
|  |  | LN | 94.3 | 91.7 | 33.9 | 53.1 | 11.2 | 1.7 | 16.7 | 16.0 |
|  |  | SPL | 88.9 | 97.2 | 14.7 | 37.6 | 0.6 | 47.1 | 8.3 | 24.7 |
|  | #340 | BM | 50.9 | 3.91 | 66.3 | 24.1 | 3.5 | 6.1 | 0.5 | 0.0 |
|  |  | LN | 99.8 | 70.4 | 63.5 | 31.1 | 3.3 | 2.1 | 3.0 | 1.0 |
|  |  | SPL | 96.6 | 22.1 | 54.6 | 33.2 | 5.0 | 7.2 | 1.8 | 0.9 |
|  | #344 | BM | 67 | 62.7 | 62.0 | 24.0 | 13.7 | 0.4 | 17.0 | 3.1 |
|  |  | LN | 97.1 | 84.0 | 52.8 | 36.3 | 9.4 | 1.5 | 18.9 | 8.4 |
|  |  | SPL | 91.6 | 77.1 | ND |  |  |  |  |  |
|  | #347 | BM | 16.5 | 37.7 | 75.1 | 21.4 | 3.0 | 0.5 | 16.2 | 1.7 |
|  |  | SPL | 67.3 | 69.2 | 67.6 | 19.8 | 8.0 | 4.6 | 16.6 | 3.1 |
|  | #349 | BM | 54.4 | 96.6 | 48.7 | 39.2 | 11.4 | 0.7 | 26.8 | 4.9 |
|  |  | SPL | 63.6 | 98.2 | 30.7 | 48.8 | 3.6 | 17.0 | 14.3 | 3.4 |
|  | #399 | BM | 51.7 | 77.4 | 67.0 | 20.5 | 12.0 | 0.6 | 16.5 | 2.3 |
|  |  | SPL | 81.2 | 86.7 | 49.6 | 37.3 | 3.8 | 9.2 | 12.1 | 3.5 |
|  | #404 | BM | 49.5 | 85.0 | 91.2 | 5.5 | 1.1 | 2.2 | 10.2 | 0 |
|  |  | SPL | 18.2 | 75.4 | 59.2 | 16.5 | 4.1 | 20.3 | 23.9 | 0 |
|  | #406 | BM | 76.4 | 96.7 | 78.9 | 13.6 | 6.2 | 1.5 | 27.0 | 1.5 |
|  |  | SPL | 60.7 | 72.4 | 62.2 | 24.3 | 2.4 | 11.2 | 15.0 | 0.8 |
|  | #407 | BM | 84.7 | 84.2 | 43.8 | 38.5 | 12.9 | 4.7 | 14.9 | 2.2 |
|  |  | SPL | 44.8 | 84.0 | 33.6 | 39.1 | 18.5 | 8.8 | 13.6 | 0.2 |
|  | #420 | BM | 15.5 | 93.0 | 79.6 | 14.1 | 3.6 | 2.8 | 10.4 | 0.7 |
|  |  | LN | 93.1 | 69.9 | 26.5 | 32.4 | 32.5 | 8.6 | 0.2 | 0 |
|  |  | SPL | 24.6 | 84.4 | 40.1 | 32.5 | 13.0 | 14.3 | 17.8 | 0 |
|  | #455 | BM | 75.9 | 69.2 | 54.9 | 14.4 | 3.7 | 27.0 | 5.0 | 0.3 |
|  |  | SPL | 66.2 | 63.5 | 46.6 | 28.7 | 1.6 | 23.0 | 6.7 | 2.2 |
|  |  |  |  |  |  |  |  |  |  |  |
| M22 | #402 | BM | 20.8 | 23.0 | 68.8 | 2.7 | 2.7 | 25.9 | 2.6 | 0.2 |
|  |  | SPL | 51.4 | 17.6 | 28.1 | 5.5 | 1.4 | 65.0 | 0.9 | 0.3 |
|  | #409 | BM | 30.4 | 22.7 | 70.7 | 0.7 | 0.7 | 27.8 | 1.0 | 0.2 |
|  |  | SPL | 32.5 | 22.1 | 31.0 | 9.2 | 1.5 | 58.3 | 1.3 | 0.8 |
|  | #414 | BM | 8.0 | 16.7 | 16.7 | 16.7 | 0 | 66.7 | 2.8 | 2.8 |
|  |  | SPL | 37.5 | 8.3 | 45.4 | 11.5 | 1.5 | 41.5 | 0.5 | 0.3 |
|  |  |  |  |  |  |  |  |  |  |  |
| Mock | #413 | BM | 10.0 | 20.0 | 57.9 | 0 | 0 | 42.1 | 2.1 | 0 |
|  |  | SPL | 25.7 | 22.4 | 35.5 | 5.2 | 4.8 | 54.5 | 0 | 0 |
|  | #422 | BM | 32.6 | 14.1 | 42.9 | 6.6 | 2.9 | 47.6 | 0.9 | 0.1 |
|  |  | SPL | 16.0 | 14.5 | 25.2 | 14.1 | 8.7 | 51.9 | 1.5 | 0.5 |
|  | #446 | BM | 26.4 | 36.8 | 47.5 | 0.9 | 2.4 | 51.0 | 3.1 | 0.3 |
|  |  | SPL | 44.0 | 9.59 | 18.0 | 4.3 | 0.6 | 77.1 | 0.1 | 0.4 |
