# Supplementary figures and images for: PDZ domain-binding motif of Tax sustains T-cell proliferation in HTLV-1-infected humanized mice
Source: PLoS Pathog. 2018 Mar 22;14(3):e1006933. doi: 10.1371/journal.ppat.1006933 (PMC5882172; doi:10.1371/journal.ppat.1006933)

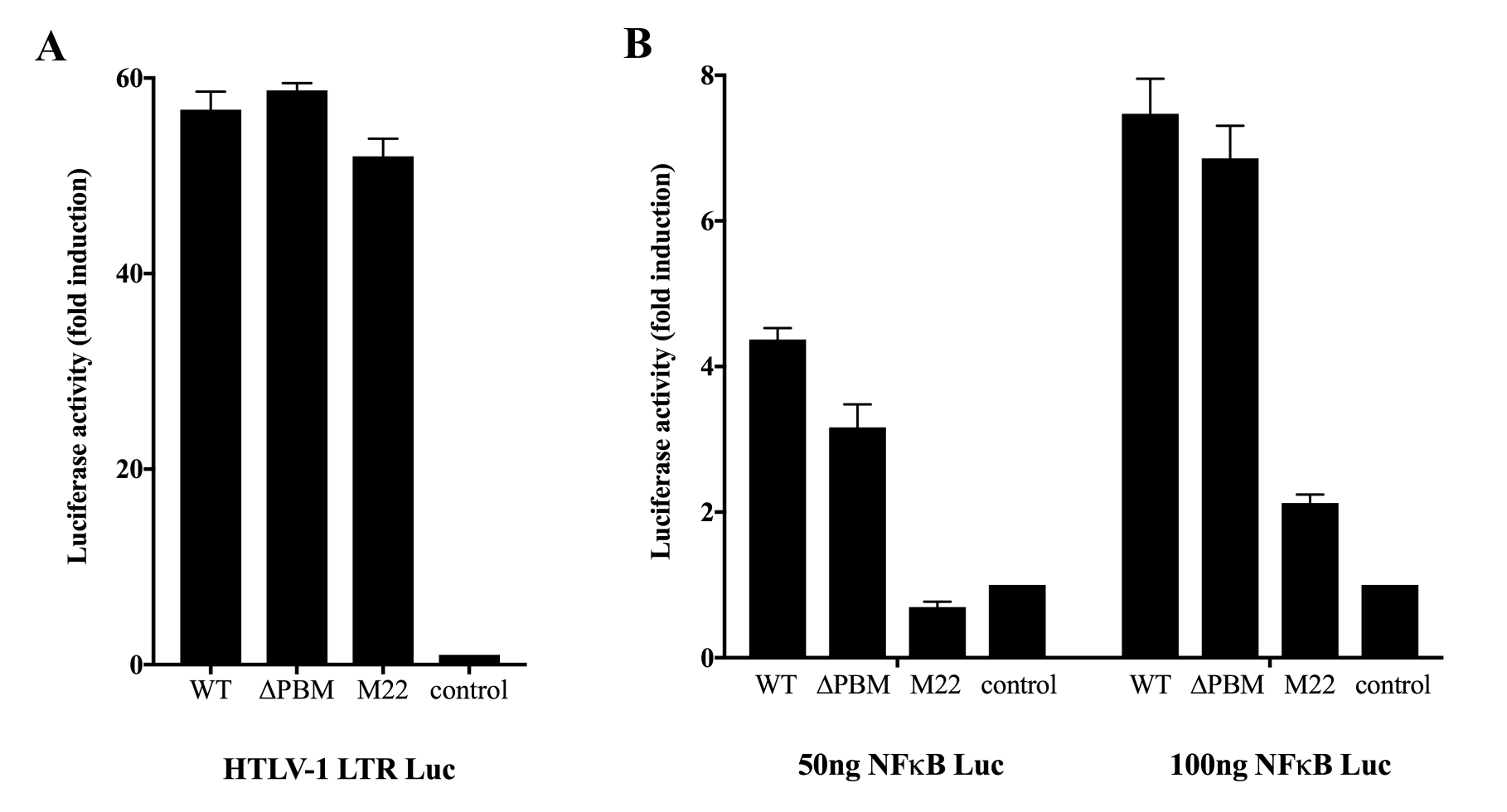

Supplement: S1 Fig — 293T cells (9 × 10^4^/ 24 well) were cotransfected with the indicated ACH plasmid (100 ng), TK-Renilla (5 ng) reporter plasmid together with the HTLV-1 LTR-luc (A), or the κB-luc (B) as calcium phosphate coprecipitates. Cell lysates were harvested 48h after transfection and luciferase activity was determined using the Dual Luciferase Assay System (Promega). The histogram presents the average fold activation over control values for 2 independent experiments in triplicate; data are presented as mean ± SEM. (TIF) [file ppat.1006933.s007.tif]

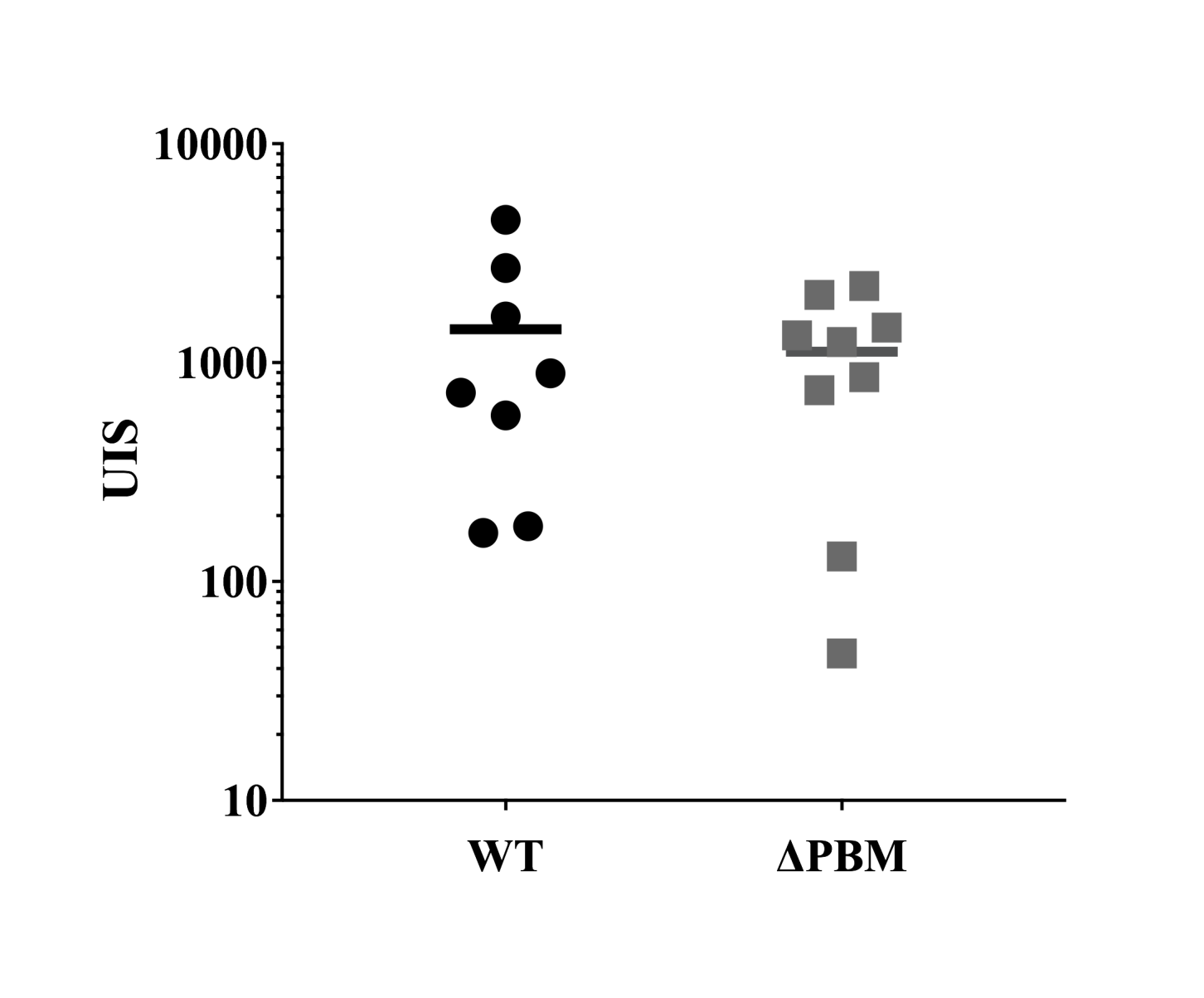

Supplement: S2 Fig — The number of independent HTLV-1-infected clones was determined by HTS clonality analysis in splenocytes (8 WT and 9 ΔPBM). Bar represents mean. Student t-test, P = 0.3021. (TIF) [file ppat.1006933.s008.tif]

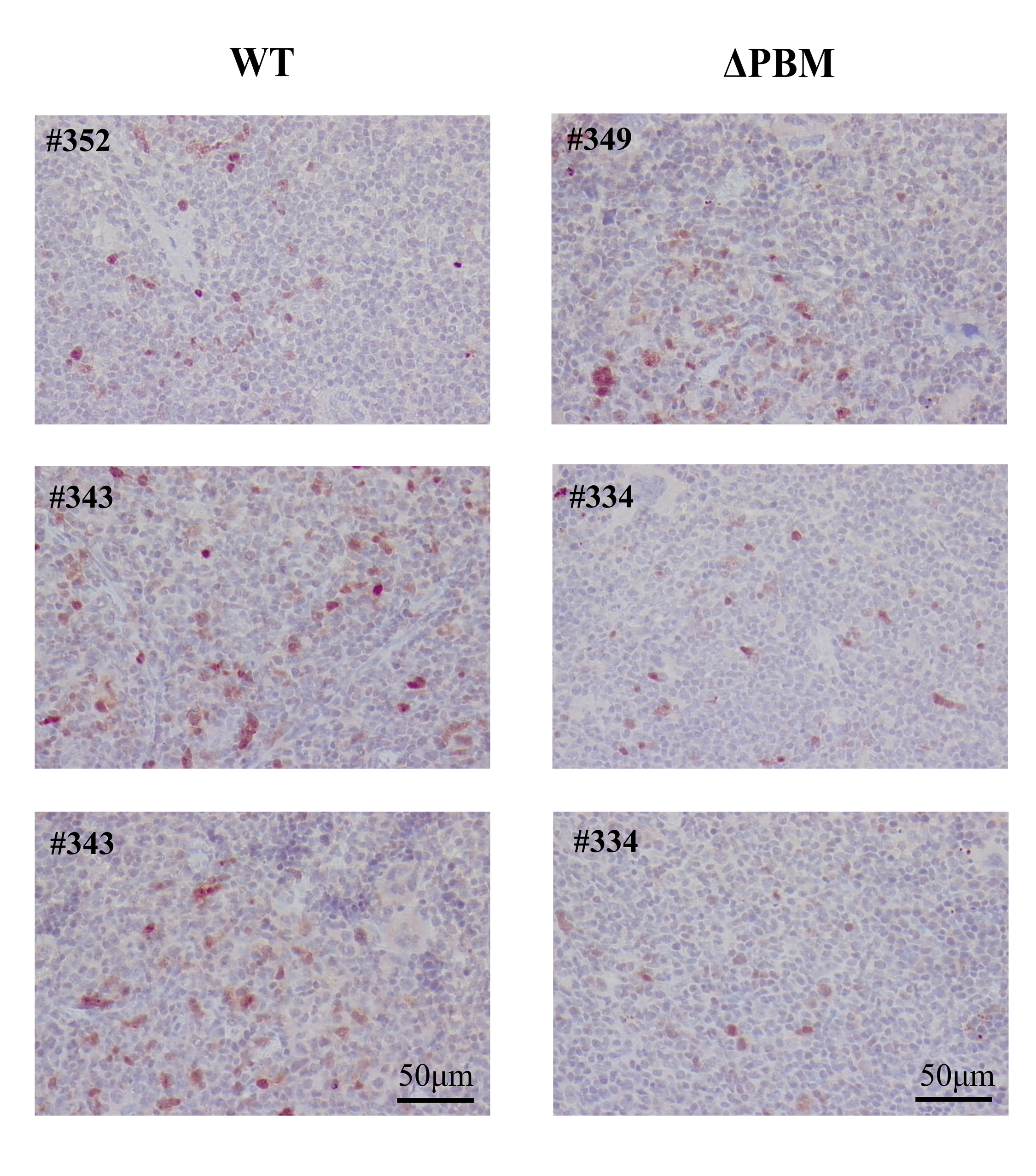

Supplement: S3 Fig — Staining with anti-Tax antibodies revealed an infiltration of T-lymphocytes with a nuclear localization of Tax. (TIF) [file ppat.1006933.s009.tif]

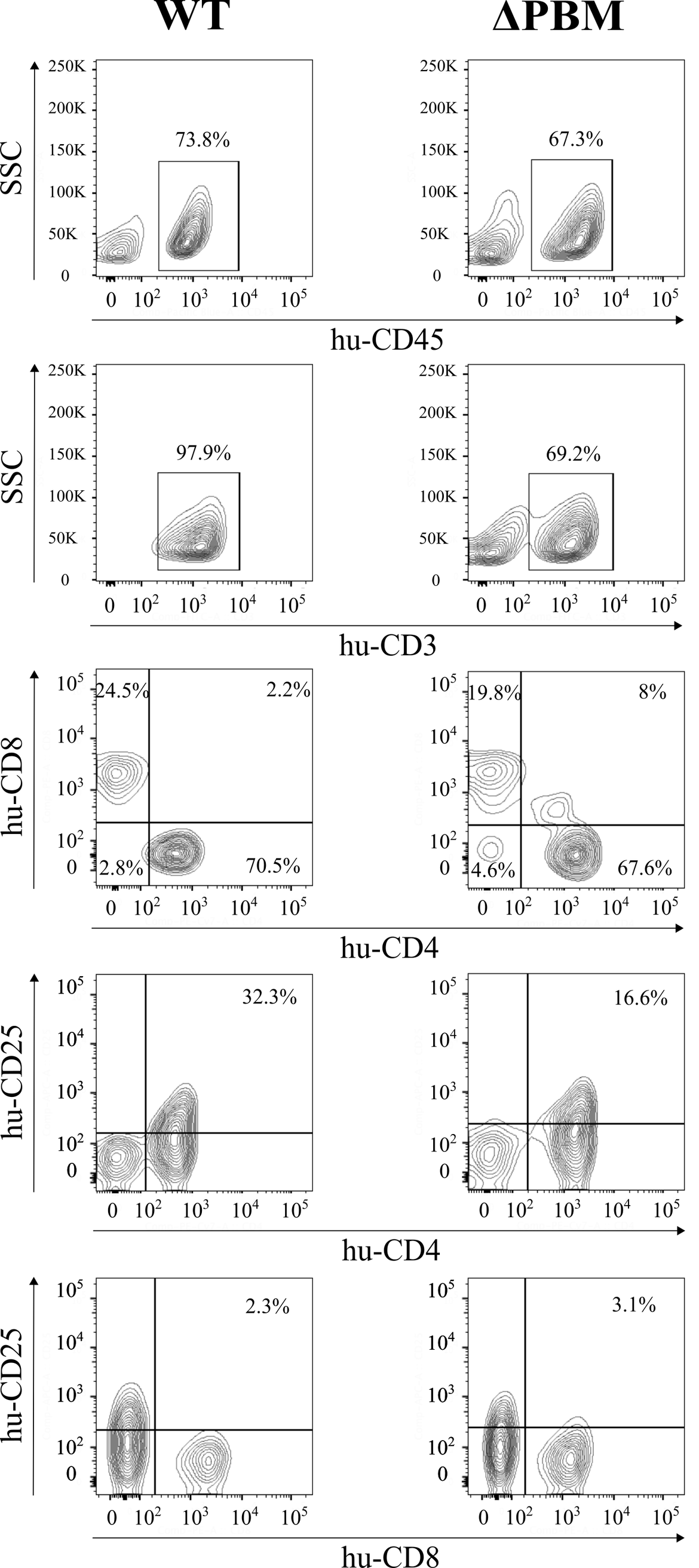

Supplement: S4 Fig — Splenocytes from WT or ΔPBM-infected hu-mice were harvested 7 weeks after infection. Representative profile for CD4, CD8, and CD25 expression on gated hu-CD3+ cells. (TIF) [file ppat.1006933.s010.tif]

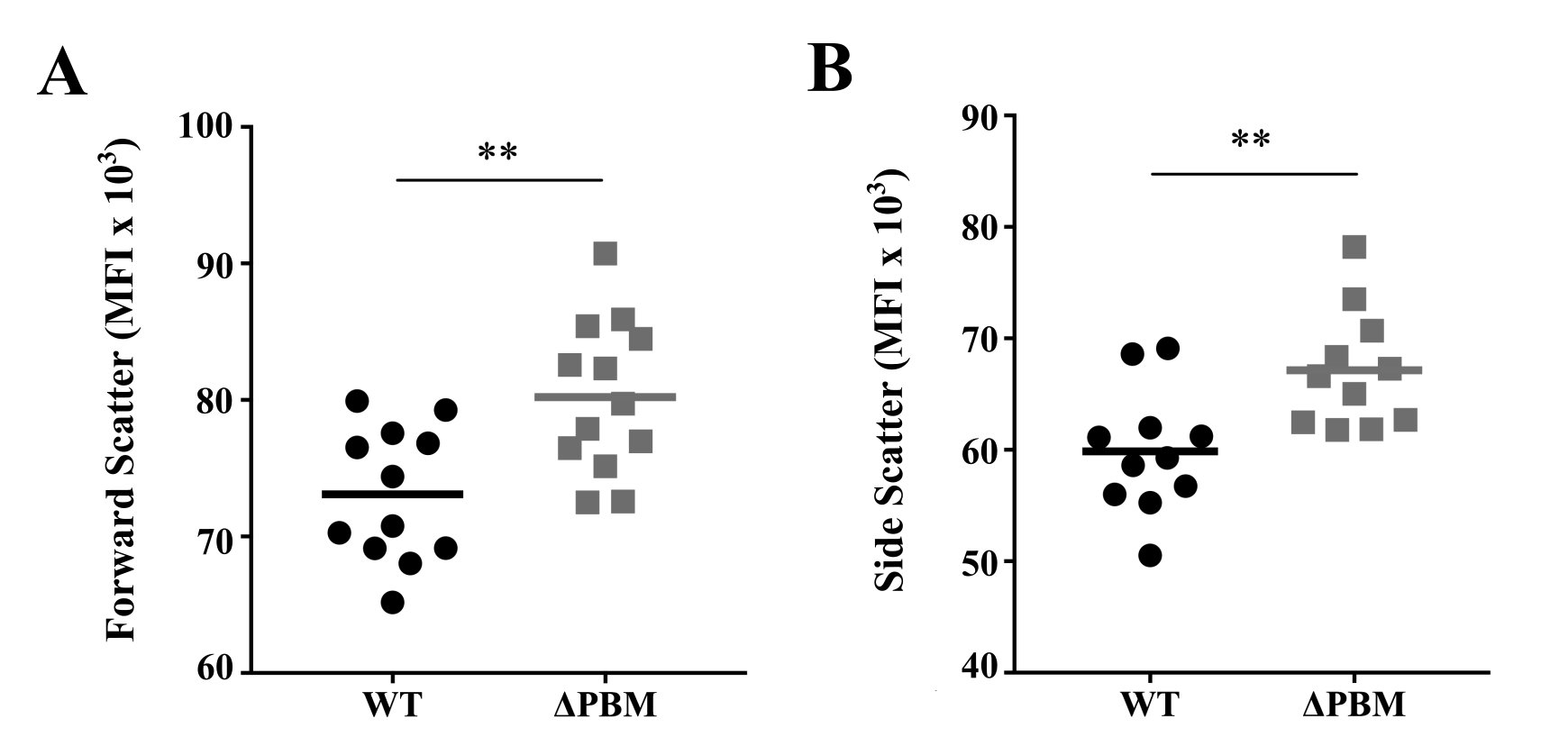

Supplement: S5 Fig — (A) Size (FSC for Forward Scatter) and (B) Granularity (SSC for Side Scatter) of CD4+CD25+ T-cells in the spleen of WT and ΔPBM hu-mice. (TIF) [file ppat.1006933.s011.tif]

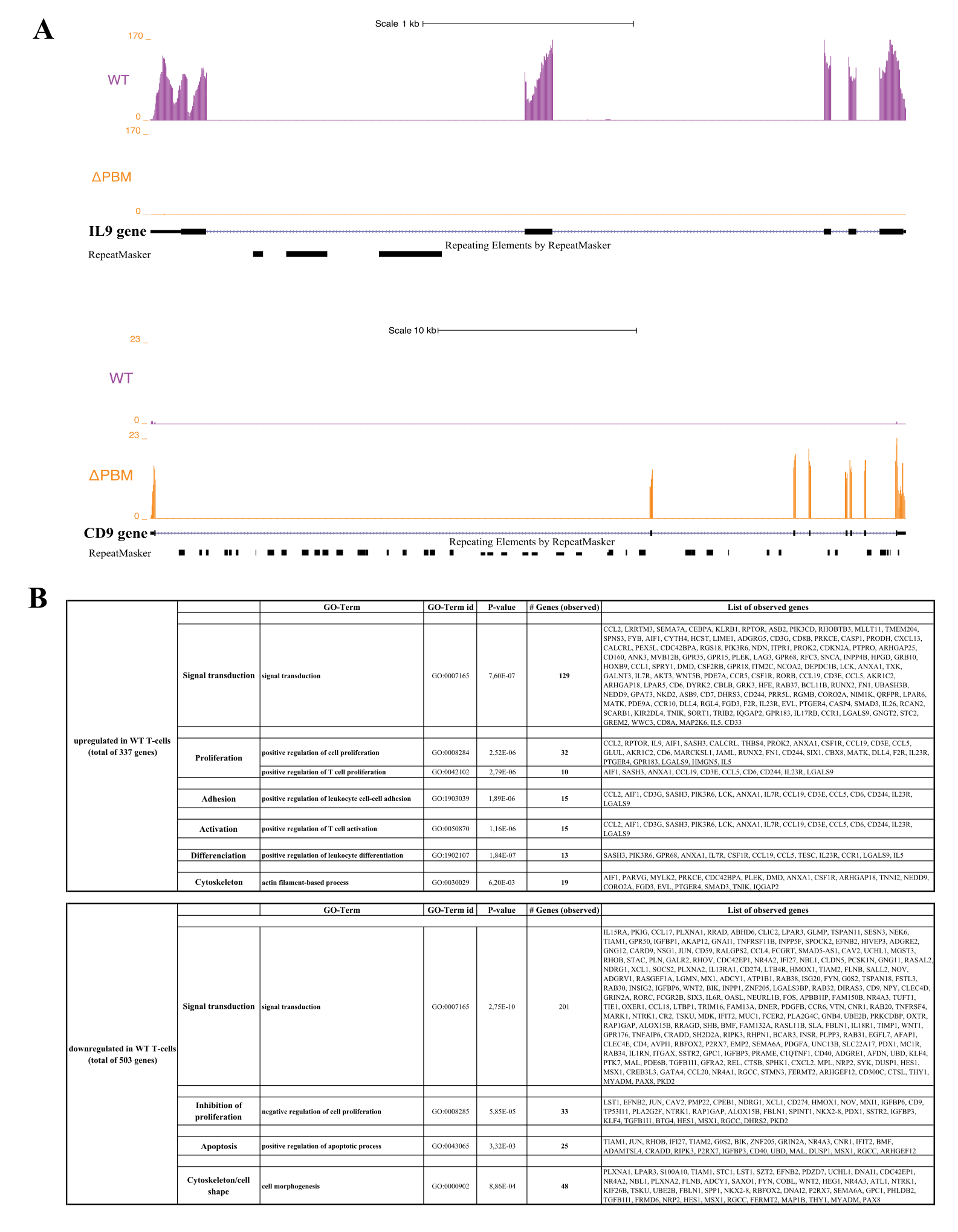

Supplement: S6 Fig — (A) Reads were mapped on the human genome (hg19). They are specific of gene exons and do not map on repeated sequences. Shown is the number of reads in the WT cells (in purple) and ΔPBM cells (in orange). (B) Detailed list of the differential expression of transcripts (adjusted P-value < 0.01) by GO annotation according to the biological process category, calculated using Genomatix GeneRanker tool. (TIF) [file ppat.1006933.s012.tif]
